# The burden of global outbreaks: Photos of the daily lives of children with congenital Zika syndrome during the COVID‐19 pandemic

**DOI:** 10.1111/hex.13717

**Published:** 2023-08-18

**Authors:** Dinara Laiana de Lima Nascimento Coutinho, Heather Feldner, Monique L. G. Coelho, Karolinne S. Monteiro, Egmar Longo

**Affiliations:** ^1^ Faculty of Health Science of Trairi Federal University of Rio Grande do Norte Santa Cruz Brazil; ^2^ Department of Mechanical Engineering University of Washington Seattle Washington USA

**Keywords:** children, congenital Zika syndrome, COVID‐19, participation, photovoice

## Abstract

**Introduction:**

In Brazil, more than 3500 children with congenital Zika syndrome (CZS) face difficulties participating in activities of daily living, which may be aggravated by health emergencies, such as the COVID‐19 pandemic. Participation could be defined as the individual's involvement in daily life situations, and participation restrictions are problems that may arise in involvement in everyday situations.

**Aim:**

To explore the daily lives of children with CZS during the COVID‐19 pandemic using photographic narratives captured by mothers and discuss possible strategies to improve participation results.

**Methods:**

In this participatory action research, seven young Brazilian mothers acted as co‐researchers using photovoice to describe the experiences of their children with CZS (from 2 to 5 years old). Also, mothers contributed to validate the contents. The research was conducted online and included the following steps: pilot study, recruitment, individualized training, sociodemographic interview, photovoice training, photo taking, focus group for contextualization, data transcription and analysis and validation of analyses by the mothers.

**Results:**

Content analysis revealed five categories that influenced the participation of the children: participation preferences, family relationships, access to healthcare, access to education and social isolation. Regarding participation preferences, mothers reported their children's desire to play with peers and family members and have autonomy. Mothers described the family environment as a happy, peaceful and safe place for the children. Lack of therapy was perceived to negatively impact the health of children; thus, treatments were considered essential for child development. Access to education included accessibility of remote education and a perceived lack of infrastructure and pedagogical preparation. Last, social isolation due to COVID‐19 directly affected the daily lives and behaviour of the children, interrupting therapies and medical appointments.

**Conclusion:**

The photos and narratives captured several aspects of the daily lives of children with CZS impacted by the COVID‐19 pandemic, reinforcing the importance of considering the negative effects of social isolation and offering education and social assistance to promote participation and integral health.

**Patient/Public Contribution:**

Consistent with a participatory action research framework, Mothers acted as co‐researchers and participated in all stages of the research, especially in validating the data analysed by the researchers.

## 1 INTRODUCTION

Latin America has endured two global outbreaks in the last decade: the congenital Zika syndrome (CZS) and the coronavirus disease (COVID‐19), both of which have significantly impacted children and families. In Brazil, children with CZS face social, medical and financial barriers, impacting their fundamental rights. A situation like the period of social distancing, necessary at the height of the COVID‐19 pandemic, faced by the whole world, making the lack of access to health and education services even greater for all children with disabilities.^1^

In 2015, during the Zika virus outbreak, the Brazilian Ministry of Health identified an increase in microcephaly cases from 0.5 to 20 per 10,000 live births.^2^ Subsequently, these young children and their families are experiencing decreased access to rehabilitation services in addition to increased social isolation during critical developmental stages due to the COVID‐19 pandemic.^3^

Zika virus infection during pregnancy may trigger a cascade of neurological and sensorineural foetal changes, constituting CZS.^4^ The most severe CZS phenotype is related to maternal infection in the first 4 months of pregnancy, with severity, presentation and prognosis characterized by the infant's head circumference at birth.^5^ According to the latest epidemiological bulletin, Brazil registered 3535 cases of children with CZS from 2015 to 2020.^6^

Children with CZS present with multiple neurological, motor, hearing, or visual disabilities and also deal with a poor intersectoral support network.^7^ Access to treatment and rehabilitation services is important for proprioceptive, sensory, linguistic, cognitive, emotional and social development. However, most specialized rehabilitation centres (CER) offer limited services, compelling caregivers to travel extensively searching for care for their children.^8^ Public policies favouring equity may help address this limitation in care, especially for low‐income populations, however, this remains a significant challenge.^9^

The outbreak of COVID‐19 reached a pandemic status in March 2020,^10^ when a health emergency state was declared, and preventive measures were enforced worldwide. Some health conditions were considered a higher risk, including cerebral palsy and CZS.^11^ Children with disabilities infected with COVID‐19 showed severe clinical conditions and a worse prognosis compared to nondisabled peers. Due to the increased risk of infection for this population combined with the reduction of in‐person healthcare, rehabilitation services and recreational services offered during this uncertain time, children and families with cerebral palsy and CZS have experienced even greater social isolation and reduced access to care.^12^, ^13^, ^14^ In fact, social restriction is one of the major impacts of health emergencies, especially for children with disabilities who already face limitations and social barriers.^15^ However, equity in care positively influences the well‐being of children with disabilities, their families and the entire community during emergencies.^16^, ^17^ Therefore, with an aim of ensuring access and equity as fundamental human rights for everyone during the pandemic, strategies are needed to understand and minimize the consequences of social isolation, loss of services and other changes in the daily lives of these children and families.^18^

One of the ways to target a deeper understanding of the experiences of children with disabilities and their families during the pandemic is by examining the construct of meaningful participation. Participating in activities of daily living is a human right and a key component for good physical, mental and social conditions for children with disabilities.^19^, ^20^ Initially defined by the World Health Organization's International Classification of Functioning, Disability and Health (ICF) as involvement in life situations, participation is now part of related constructs that include two elements: attendance and involvement.^21^ Participation is also a major outcome of interest in child rehabilitation. A viable and effective strategy to assess the participation of young children according to the perception of parents is the photovoice method.^22^

Photovoice is a method within the participatory action research (PAR) framework that evaluates a phenomenon or experience of interest from the point of view of participants as co‐researcher, through their own photographs and narratives.^23^ Photovoice is a useful tool to document and understand concepts affecting a particular group of people, such as social determinants of health, the influence of family and friends, adherence to rehabilitation programs, service quality and availability of care systems for specific groups.^24^ Through the images and narratives used to describe their experiences, participants act as agents socially transforming the reality of the community and facilitating the understanding of information on relevant topics.^25^, ^26^ Photovoice is versatile and effective in assessing the needs and understanding the experiences of people with disabilities.^22^ However, some authors suggest more studies using this technique.^27^

Considering the lack of research using this framework, and the need to more specifically understand the participation of children with CZS experiencing social isolation due to COVID‐19, this study's aims were twofold: (1) Explore the daily lives of young children and their families via photovoice, and (2) Discuss and evaluate strategies to improve participation in this population during a global pandemic.

## 2 METHODS

This study used a combination of PAR and qualitative research methods. Photovoice was employed with mothers as co‐researchers to photograph and contextualize the daily lives of children with CZS.^28^ Focus groups were then conducted to discuss and evaluate strategies to improve the participation of these children during the pandemic. Qualitative approaches, including online focus groups, are also beneficial methods for engaging with stakeholders that share similar experiences to elicit similar and divergent perspectives as well as generate new ideas in a group dynamic.^29^

### 2.1 Participants

Mothers of children with CZS were recruited from rehabilitation centres in the state of Rio Grande do Norte. The participants were involved in other projects, attended these centres and were already known by one of the researchers, they were invited by telephone call in which they received brief information about the study, and data were collected and analysed remotely.

The targeted sample size (6–10 participants) was intentional, and based on recommended group sizes in the literature that are generally adequate for deepening social relationships and ensuring meaningful contributions from all group members in qualitative studies.^30^ The chosen sample size has also been shown to be effective in collecting information and discussing photos used during photovoice.^31^

### 2.2 Procedures

**Figure 1:**
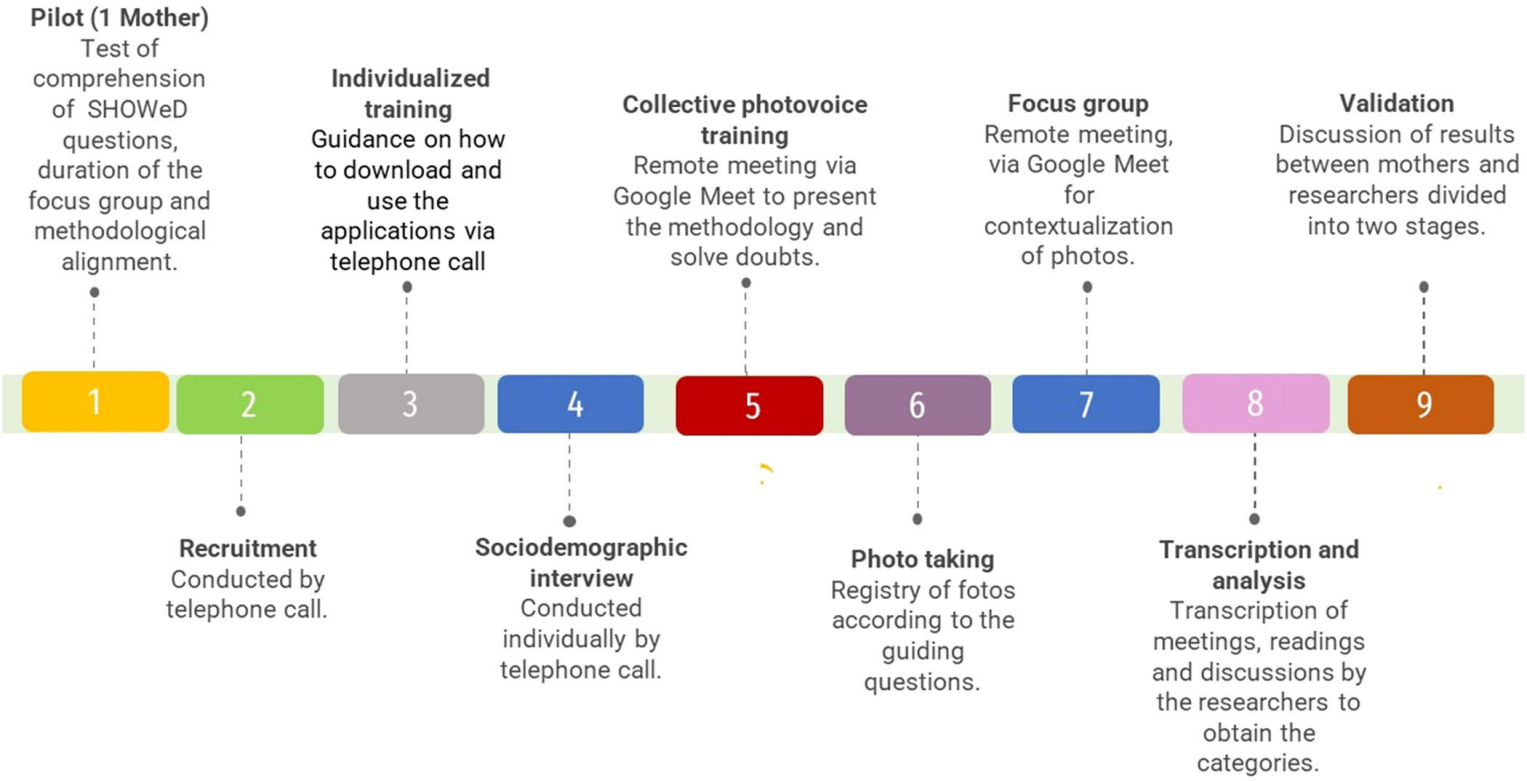
Timeline for photovoice application.

Data collection occurred between September 2020 and June 2021, following a nine‐step script from recruitment to validation of results (Figure 1).

A pilot photovoice study was carried out to explore the feasibility of planning, the effectiveness of executing the method and the identification of possible methodological blocks to participant adherence.^32^ It included a mother of a 6‐year‐old child with CZS, who participated individually in the training stage, using the same methodology and applications intended for the broader participant group. The pilot aimed to explore the understanding, and relevance of the questions selected for the focus group, and estimate the duration of the interview, for this reason, it was carried out with the purpose of aligning the methodology. Therefore, the data referring to this participant were not considered in the analysis.

Mothers in this study's cohort used their cell phone cameras to photograph important moments, people and places, including themselves and their children. Mothers had 45 days to take photos that visually demonstrated their answers to six guiding questions (Table 1). Mothers were asked for between 6 and 18 photos, totalling 1 to 3 images for each question. After selection of their individual photos, each mother's selected photos were shared within the full participant group, and the mothers voted individually to select the five photos that best represented each category.

**Table 1:** Overview of the content explored in group stages.

Stage	Content
Collective photovoice training	Presentation of research objectives, details about the photovoice applications, and execution calendar.
	Explanation of the informed consent form and the authorization for the use of image, voice and/or likeness using google forms.
	Instructions for capturing photos and emphasizing the guiding questions: 1—What makes my child happy? 2—What makes my child sad? 3—What my child likes to do? 4—What does my child not like to do? 5—What facilitates the daily life of my child in this social isolation? 6—What hinders the daily life of my child in this social isolation?
Focus group	Exhibition and reflection on selected photos
	Discussion about the photos using an adapted SHOWeD methodology (Liebenberg, 2018): **S**: What do you see in this photo? What does it represent to your child? **H**: What happened or is happening in the photo? Briefly describe it. **O**: How does this relate to your life? **W**: Why does this happen? **E**: What could this image represent to other people? What did your child experience (feelings, thoughts and sensations)? **D**: What can we do about it?

Following the photo taking, a focus group was conducted with the mothers. Dialogues on predetermined topics occurred in a focus group through the use of a semi‐structured guide for approximately 90–110 min (Table 1). During the focus group/interview, the participants exposed, shared and discussed the photos, allowing a deep reflection on the meaning of each moment in the children's daily lives. The focus group was led by a moderator (responsible for facilitating the dialogue and ensuring all participant voices were heard), and an observer (responsible for note‐taking, capturing nonverbal information and offering support).^33^, ^34^ If participants became unavailable for the focus group, individual interviews were offered at a later date.

Researchers use an adapted version of the SHOWeD methodology (Table 1) to guide the contextualization of photos taken using photovoice; it includes five predetermined questions to identify the problem demonstrated or situation exposed in the photo and identify or create strategies to improve them.^35^ Problem identification facilitates the codification of themes, whereas strategy identification aims at awareness of and action for social change. This set of questions may be used directly or indirectly to guide a discussion or investigation.^36^, ^37^

The interviews and the focus group were audio and video recorded in full with permission to enable further analysis.

### 2.3 Content analysis

According to Leal and Henriques,^38^ the process of content analysis first begins in the focus group, which contextualized the photos, created a debate and allowed the exchange of experiences. Data transcription in audiovisual format was cross‐referenced with digital recordings to ensure information accuracy. This stage was performed by an independent researcher and verified by another researcher present in both stages. Transcripts were then reviewed to cluster similar experiences into categories.

Two researchers independently analysed all data, followed by a group discussion of preliminary results among the research team. The content was analysed using an inductive approach from themes extracted from the data, according to Sposito et al.^39^ and Leal and Henriques,^38^ comprising six steps: familiarization with the data, creation of initial codes, search for themes, revision, definition and naming of themes and report writing. As co‐researchers, the mothers participating in the study collaborated in the final analysis stage, validating the established categories. The first validation was conducted remotely and individually, in which the mothers chose which category most closely aligned with the sentences and photos exposed. A researcher presented the opinion of the researchers, and in case of divergences, the mother decided the category.

Two categories overlapped, requiring a second validation, in which a booklet was developed with the photos selected by the mothers in the reorganized categories. The mothers received the material via message and were asked to read it and answer whether they agreed with the reorganization. All mothers participated in this stage and sent responses to the main researcher. All information was discussed among researchers to ensure rigour in content analysis and data reliability.

### 2.4 Trustworthiness

All researchers have experience with qualitative research, three are senior researchers, and one has previous experience using photovoice. Two authors already knew the participants, but the focus groups and interviews were conducted by research assistants who had no previous relationship with the mothers, to minimize the possibility of bias. The same procedure was adopted in the transcription, collection and analysis of the contents. Table 2 presents strategies used to minimize the risks of bias at various stages of the research.

**Table 2:** Strategies used to establish rigour.

Strategy	Method	Details
Credibility	Triangulation by different researchers	Content analysis was by two researchers.
	Member verification	Interview summaries were provided to all participants during data collection. A summary of the categories was presented to all participants during the content analysis.
	Interrogation in pair	Three researchers discussed the thematic analysis.
Transferability	Rich description of participants	Demographic information about the participants was collected.
Reliability	Dense description of research methods	A detailed description of the methods was provided for study replication.
	Peer interrogation	As above.
Confirmability	Triangulation of different researchers	As above.

### 2.5 Ethical aspects

This research was submitted and approved by the research ethics committee of the Faculty of Health Sciences of Trairi from the Federal University of Rio Grande do Norte (opinion number: 4,216,560). Mothers signed the electronic informed consent and assent forms and the authorization for the use of image, voice and/or likeness. Pseudonyms were used to protect confidentiality.

## 3 RESULTS

### 3.1 Participants

Seven mothers of children with CZS who are followed at the Anita Garibaldi CER and the physical therapy service of the Faculty of Health Sciences of Trairi from the Federal University of Rio Grande do Norte participated in the study. One mother completed the pilot study, and six participated as a study cohort. Children were aged 3–5, and mothers were 18–36 years. Four mothers participated in the remote focus group to contextualize the photos, and two performed this step individually and remotely due to illnesses. All mothers were Brazilian, the primary caregivers and lived in different cities of Rio Grande do Norte (Table 3).

**Table 3:** Sociodemographic data.

Mother						Child				
Pseudonym	Age	Marital status	Income	Education level	Housing area	GMFCS	Age	Sex	Attends school	Uses a wheelchair
Daniela	34	Married	Four or more minimum wages	Postgraduate	Urban zone	V	5	Male	Yes	Yes
Julia	36	Married	Up to one minimum wage	High school	Urban zone	IV	5	Male	No	No
Irece	24	Single	Up to one minimum wage	High school	Urban zone	V	4	Female	No	Yes
Luisa	18	Married	Up to one minimum wage	Incomplete elementary school	Urban zone	V	5	Female	No	Yes
Marta	27	Married	Up to one minimum wage	Elementary school	Urban zone	V	5	Female	No	No
Patrícia	34	Single	Up to one minimum wage	Incomplete high school	Urban zone	V	2	Male	No	No
Pilot	33	Married	Up to one minimum wage	High school	Urban zone	IV	4	Female	Yes	Yes

Abbreviation: GMFCS, Gross Motor Function Classification System.

Photographs were taken at home or selected from the cellphone image gallery of the mothers due to social isolation as a preventive measure against COVID‐19 (recommended by the World Health Organization).

The six mothers sent a total of 73 photos. During the subsequent focus group, mothers presented between 6 and 18 photos and discussed their meaning (some had only one photo per question, and others had the maximum 3 photos requested). At the end of the focus group, mothers individually voted on which photograph best represented what had been discussed in the focus group, which fell into five main categories. This voting process made it possible to reduce from 73 to 5 photos, retaining what was perceived as the most impactful, single photo representing each category from the discussion. Subsequently, the validation procedure of the categories/photos was carried out.

Content analysis revealed five categories that represented aspects of participation of children during social isolation: participation preferences, family relationships, access to health and developmental supports, access to education and social isolation. The findings and selected photos are presented below, with additional quotes from the mothers included to support each thematic category.

#### 3.1.1 Participation preferences

In response to the photos selected in this category, the mothers described situations regarding independence, autonomy, respected wishes and the desire to play with other children and family members to represent the preferences of children. For example, Julia reported:

He expresses a feeling of joy and a little bit of independence by being able to stand at the gate. In this photo, he was holding on to the gate; it is the moment I put on the orthosis, and he stands up; he was looking at the street thinking he was going out because he is crazy about the street. (Boy, 5 years old, Gross Motor Function Classification System [GMFCS] = IV, Julia)

Photo 1: Autonomy as a participation facilitator.

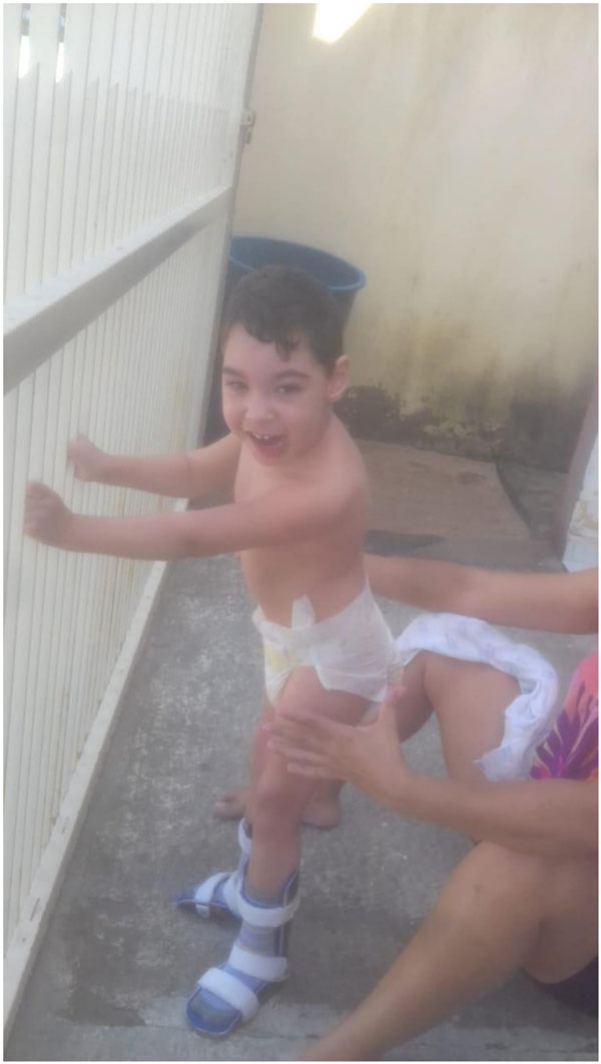

*Source*: Julia, mother of Daniel.

Julia describes how the use of orthoses supports her child's standing, which in turn reflects joy, a sense of independence, and a different perspective of everyday life. Overall, four mothers reported satisfaction regarding autonomy.

I think he likes it when we let him do something. (Boy, 5 years old, GMFCS = IV, Julia)

She just wants things her way. (Girl, 5 years old, GMFCS = V, Luisa)

I think every time we do what he likes, he shows he is happy, satisfied. (Boy, 5 years old, GMFCS = V, Daniela)

When playing, the children reacted with a smile, body expressions (moving arms and legs), and shouts of happiness that showed engagement in the activities. Two mothers reported that being alone displeased the children. To express loneliness, children cried and screamed until someone appeared, as shown in the following report:

He dislikes being alone. He does not want to be alone at any time. If he is alone, he cries, he complains … that is why I posted this picture of him in bed; that is the only time we know he does not like it. Every moment of the day, we try to involve him with the family, at lunchtime, at dinner … We always put him close; wherever we are, he is with us. (Boy, 5 years old, GMFCS = V, Daniela)

Julia added:

In this photo, he is alone, sitting. He does not like to be alone for too long. In the photo, he is happy because he saw I was coming back to get him. (Boy, 5 years old, GMFCS = IV, Julia)

#### 3.1.2 Family relationships

Mothers reported the family environment as a happy, peaceful and safe place for the children. Daniela declared: ‘The company of his father, his sister, and I is what makes him have a better development; it is what makes him play and smile’. Moreover, the distancing of family members often created negative experiences, as seen with one child who was separated from her father for a few days because of work:

In this moment her father was leaving to work, and I took the picture. She was crying because her father left the house. I think she imagines he will not come back home. (Girl, 5 years old, GMFCS = 5, Marta)

Children constantly repaid the affection offered by their families with smiles, looks and conversations, as Patrícia reported: ‘He feels loved and responds to affection with this smile, with his look’ (Boy, 2 years old, GMFCS = V). Similarly, Irece explained:

This photo is when it was time to sleep, and she wants to keep talking. She is expressing a feeling of wanting attention, for me to pay attention to her, to play around. (Girl, 4 years old, GMFCS = 5)

Regarding engagement in activities, the mothers highlighted that the children experienced participation by being involved in activities with other children or a family member.

He likes to play, likes to be at home with his family. (Boy, 5 years old, GMFCS = IV, Julia)

Her little cousin feeding her is what makes her happy, her cousin coming to our house and feeding her. (Girl, 4 years old, GMFCS = 5, Irece)

He shows us a happy feeling for being with family, that feeling of peace, of tranquility. The photo demonstrates the reality of his life. (Boy, 5 years old, GMFCS = V, Daniela)

Photo 2: Family relationships.

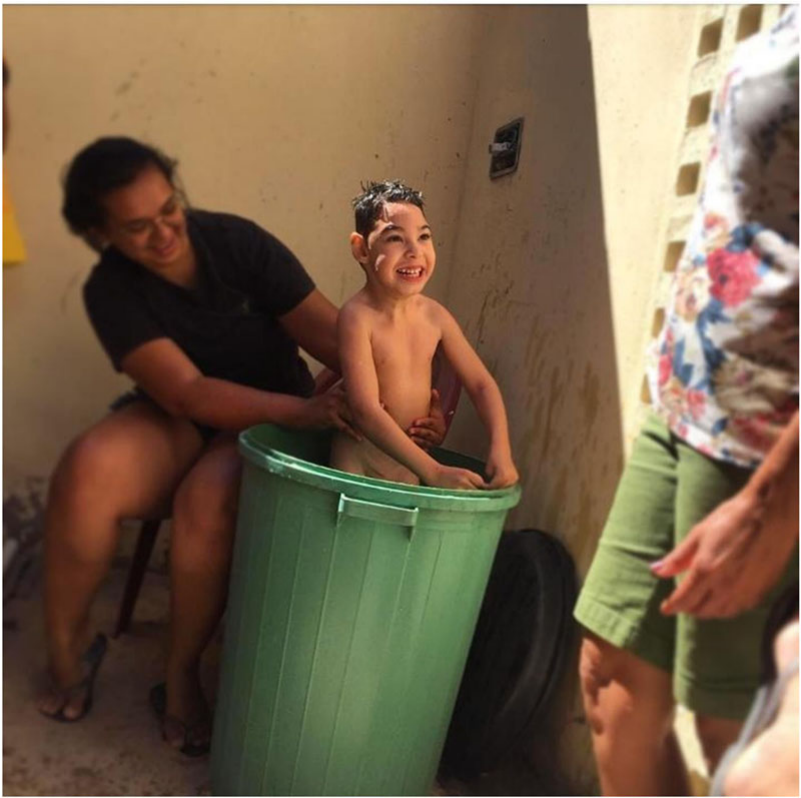

*Source*: Julia, mother of Daniel (2020).

#### 3.1.3 Access to health and developmental supports

Before the pandemic, the children had a busy routine, with many trips for treatments and regular medical appointments. Physical and occupational therapies were mentioned in all of the mothers' statements as essential to child development. The absence of one or both therapies may have impaired the health and development of the children, as Patrícia reported:

He started physical therapy when he was one‐month‐old, then we came to the Northeast, and we lost everything he had achieved due to lack of physical therapy. (Boy, 2 years old, GMFCS = V, Patrícia)

Marta added: ‘Now, in the pandemic, the treatment is more difficult because she does not have physical therapy, which helps a lot’ (Girl, 5 years old, GMFCS = 5, Marta). Therefore, we observed that treatment disruption due to the COVID‐19 pandemic delayed the evolution of treatments.

Photo 3: Physical therapy as a symbol of access to health.

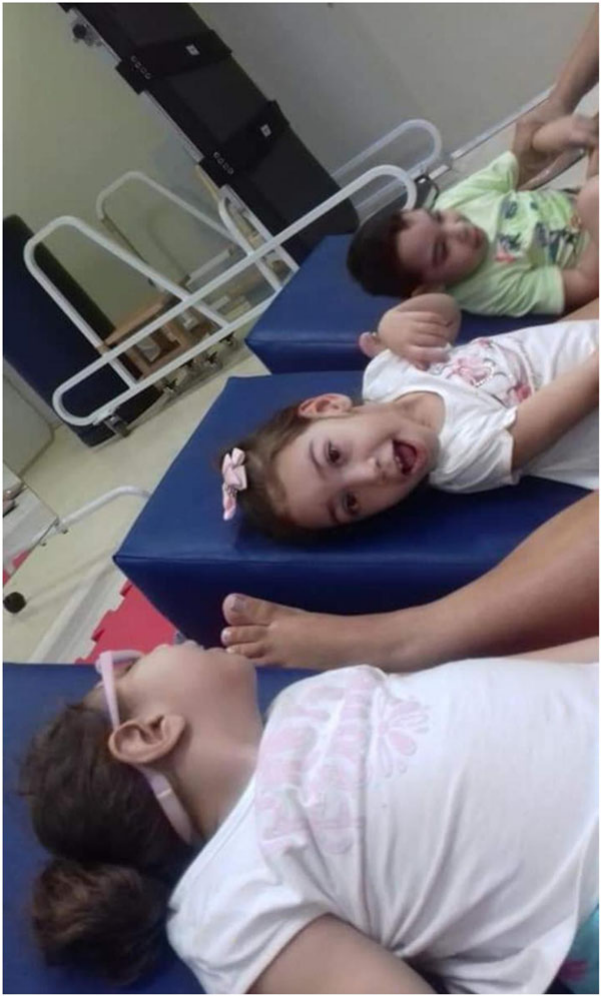

*Source*: Marta, mother of Maria Clara (2020).

Another key developmental support presented by Patrícia was the use of an appropriately fitting orthosis: ‘His leg is very bent. It is one on top of the other. He tries to move his leg but cannot, maybe because of the leg weight and because the upper leg is hurting the bottom leg. It appears [the orthosis] is weighing him down, bothering him, squeezing him … like he wanted to take that thing off’ (Boy, 2 years old, GMFCS = V, Patrícia). This mother's perception was that an unadjusted or poorly fitting orthosis may negatively impact her child's well‐being and lead the child to resist this therapeutic resource.

Four children used a wheelchair as an assistive device for locomotion; only one was acquired with private family resources. Luisa shared that the public service provided the wheelchair: ‘We got her wheelchair from City Hall. We could never get it from the CER…. since 2018, it never worked out’ (Girl, 5 years old, GMFCS = V, Luisa). In contrast, another mother vented:

He still has not received the chair due to lack of interest from the government, since I already requested it at the CER three years ago and we never received it. I lack the money; I cannot afford to buy it; it costs five thousand reais. (Boy, 5 years old, GMFCS = IV, Julia)

Regarding public resources, two mothers mentioned receiving assistance from City Hall. Irece said: ‘Almost every day she went to physical therapy using the City Hall car’ (Girl, 4 years old, GMFCS = 5, Irece).

Patrícia informed:

Before the pandemic, we would go out a lot to look for treatments, physical therapy, medical appointments, and every benefit he is entitled to … so every week we had something to do and many exhausting trips … however, with the pandemic, the development regressed a lot. He can no longer control his neck due to a lack of physical therapy. Previously, he could take a few steps; he would turn around more. Now, what he has gained, he has already lost … his leg is atrophying a lot and is very crooked compared to the other. (Boy, 2 years old, GMFCS = V, Patrícia)

#### 3.1.4 Access to education

Although education is a right of every citizen, mothers reported that educational environments were commonly unprepared to receive children with disabilities, impacting their participation in school activities. Luisa reported mixed attitudes of school supervisors, both friendly or negative: ‘I enrolled her in a daycare center, but she stayed less than a month because they put her in a class of two‐year‐olds. [The children] cried a lot; she would get irritated and could not adapt. I noticed that the teacher did everything in her power for her to stay at school. She would call and get in touch to convince me to take her to school. On the other hand, the principal did not seem to mind the presence of my daughter and would not even bother to prepare the school to receive her’ (Girl, 5 years old, GMFCS = V, Luisa).

The mothers' perspectives highlighted that school inclusion goes beyond physical structure; it involves training professionals and promoting disability awareness among people engaged in these environments. Few schools promote inclusion and prevent ableism; however, some were adaptable and willing to work with families, especially as online learning increased during the COVID‐19 pandemic, as Daniela described:

‘As all schools are performing online activities, the teacher sends the activity, and we take time during the day to do it with him. He likes this movement of painting, us following his motor coordination, so much so that his left arm is very smooth regarding movement. Here in the city, we have a teacher specialist in special education. I reached out to her and talked to her, and she was willing to work with him regarding his needs’. (Boy, 5 years old, GMFCS = V, Daniela)

Photo 4: Remote education during the pandemic.

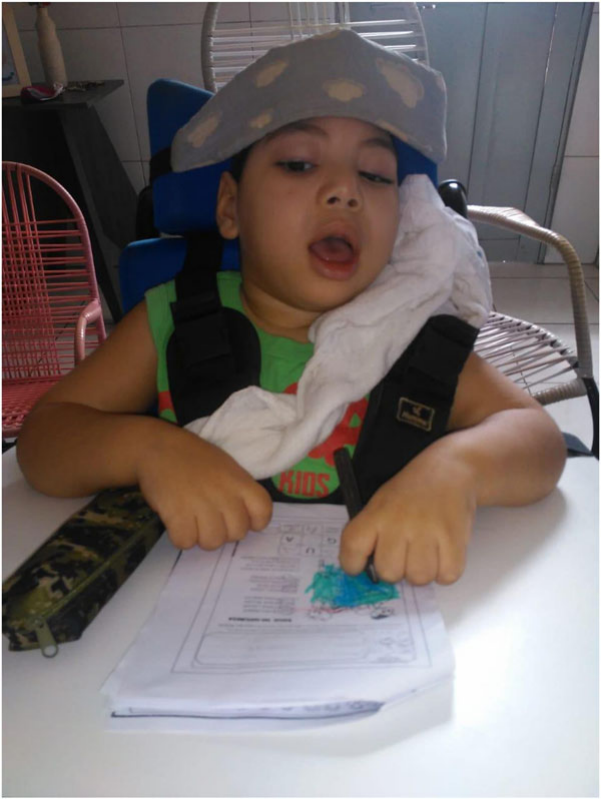

*Source*: Daniela, mother of Leonardo (2020).

#### 3.1.5 Social isolation

Though social isolation brought barriers to many families, three mothers reported that children actually enjoyed more time to stay home with family members during social isolation.

We used to go out a lot to physical therapy, medical appointments, and could not enjoy our time together. You can see that she was happy and smiling to be with me. (Girl, 4 years old, GMFCS = 5, Irece)

After the pandemic, he stayed more at home and spent more time with his brothers and family. (Boy, 2 years old, GMFCS = V, Patrícia)

With the pandemic, he has more time to enjoy at home, play, do physical therapy. (Boy, 5 years old, GMFCS = IV, Julia)

Although children had more time to be with family members, the care interruption during the pandemic harmed the development of some children, as described in the *Access to Health and Developmental Supports* category. Mothers indicated that the lack of access to health services led to technological improvements such as telerehabilitation programs, as reported by Julia and Luisa:

The physical therapists gave us some booklets with guidelines, and he was being accompanied through video call on WhatsApp. They would send exercises that we would do at home. (Boy, 5 years old, GMFCS = IV, Julia)

Until a month ago, the physical therapist would send videos explaining what to do at home. The videos were small, and easy to understand, with a few different tasks each week. They were exercises teaching how to roll over and control the trunk. Everything she would do at the school clinic was explained in the video; it was like continuing the treatment. (Girl, 5 years old, GMFCS = V, Luisa)

Part of social isolation also involved masks or face coverings, which impacted the social responses of some children. For example, during the discussion about the needed care during the pandemic, Daniela described how her son negatively reacted to the mask for the first time:

In this photo, we were in Fortaleza, and I had never put a mask on him. He needed to have the mask on to enter the hospital Sarah. When I put it on, he turned his head to the side, and no one could take his head out of that position. He only adjusted his head when he took off the mask. It is one of the things he is most angry about: a mask around him. (Boy, 5 years old, GMFCS = V, Daniela)

Another negative point identified by mothers was the behaviour change they observed in their children. Due to being at home for longer periods, some children became more introspective and sad:

When the pandemic is over, I think it will be difficult for her to return to her routine with her tasks. Lately, she dislikes going out or interacting with other people. Nowadays, she likes to stay in her corner … before, she used to like to go for walks. (Girl, 5 years old, GMFCS = V, Luisa)

His life became more difficult in this daily isolation because he used to go out a lot, and we would take him everywhere we went … Now he only has contact with his family, the three of us at home together with him … I think that was the part he felt the most: he would go out with us a lot, and now we are trapped inside the house. When we leave, it is one or the other, but he remains inside the house. So, for him, I think this isolation part that he needed to stay home more, unable to leave or have fun, made him sadder. I think his sadness is clear when we need to go out. (Boy, 5 years old, GMFCS = V, Daniela)

What was difficult was because she has a wheelchair, and since the beginning of the isolation, she could not leave home; she could not walk around in the wheelchair. In this photo, she is sad, looking at the gate, longing for a walk. (Girl, 4 years old, GMFCS = 5, Irece)

Photo 5: The effects of social isolation.

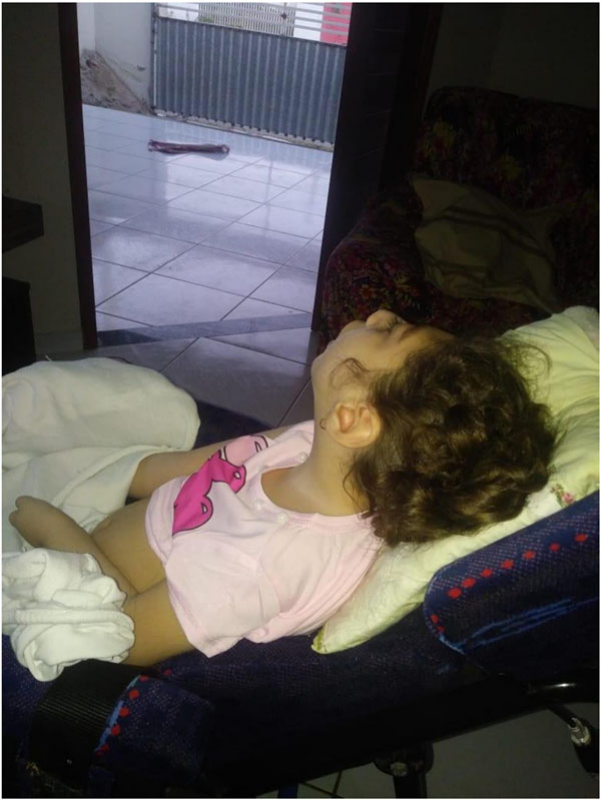

*Source*: Irece, mother of Ingrid (2020).

However, Daniela noticed the benefit of having a wheelchair during social isolation:

What made his life easier in this pandemic was having a wheelchair. In the past, he was trapped in a chair, on the couch, or on the bed. Now, even inside the house, it is easy for us to walk him around … Now wherever I go, I can take him; wherever his father is, he can take him close, and his sister also takes him wherever she needs to go. So, I think the wheelchair favored his mobility. (Boy, 5 years old, GMFCS = V, Daniela)

## 4 DISCUSSION

This PAR and qualitative study are the first to explore the daily lives and participation of Brazilian children with CZS during the COVID‐19 pandemic. Young, low‐income mothers, and primary caregivers of children with CZS, participated as co‐researchers, photographing the participation of their children and discussing their photos and stories during a focus group or interviews. Most children were five years old and required assistance for activities of daily living, including mobility in a wheelchair.

Using the photos discussed and co‐analysed with the mothers, we identified five categories representing the participation of children during the COVID‐19 pandemic: (1) Participation Preferences revealed what influenced the engagement of children in activities of daily living; (2) Family Relationships were expressed as a promoter of participation; (3) Access to Health showed situations of support for the developmental of children and some difficulties for mothers to guarantee what is their children's right; (4) Access to Education revealed two distinct situations between inclusive education and the lack of it and (5) Social Isolation, revealed benefits that favoured family union, but detriments in that it interrupted health treatments and hampered the socialization of children with CZS.

Mothers were able to discuss their perceptions of their children's feelings as well as participation and health barriers and lifestyle adaptations during the COVID‐19 pandemic. These findings corroborate a study by Reinchenberg et al.,^40^ in which mothers of children with CZS reported feelings of incomprehension and marginalization, and difficulties in daily life. Maternal perspectives such as these have also been shown to encourage government policy development centring on the biopsychosocial model of the ICF. For example, hearing about the lives and experiences of mothers of children with CZS has strengthened support and assistance networks for this population and may minimize social barriers to ensure inclusion and interaction.^41^

Regarding participation in activities of daily living, Santos et al.^42^ found that initiatives aimed at the active participation of the primary caregiver of children with microcephaly provided the caregiver with a greater sense of autonomy and responsibility, considering the child's needs. Photovoice is a technique that can assist in the empowerment process described in the literature. Sharing PAR research photos and narratives with the community, press agencies, social control organizations and public managers aims to further encourage parents to defend their children's rights and promote social change.^43^

Regarding family relationships, mothers described it as essential for their children to convey security, happiness and peace, being an important predictor for participation, similar to previous research.^44^, ^45^ These authors identified that economic status, family cohesion and family preference can influence the participation of children with cerebral palsy and other physical disabilities.

Further, according to Maria‐Mengel and Linhares,^46^ welcoming and caring for children and their families are essential for achieving greater functional gain in the first 4 years of children's lives. Family participation and collaboration are of paramount importance for the rehabilitation of children with microcephaly, it is an indispensable factor that favours the involvement of children with their therapy and with the professionals involved.^47^ Therapy services that are family‐centred, consider the child's needs, focus on structured play and recreation activities and on health and well‐being can support the development of children with CP.^48^

Regarding access to health care, the families in this study invested in the development of children with physical disabilities and sought opportunities for rehabilitation therapies. These findings also reflect the current literature, which indicates that empowering parents with ongoing guidance and regular follow‐up is essential to ensure a high quality of life for children with disabilities.^49^ However, the mothers' perspectives and stories shared in this study highlight the existing gap in access to health care and developmental support for children with CZS, as well as the exhausting routine of medical consultations and treatment (especially in large cities). Other authors also identified that health professionals and mothers of children with CZS report that health care was inadequate, distributed in several services, fragmented and centred in the urban area, highlighting the inaccessibility of specialized care in the Unified Health System.^50^

Some mothers also reported difficulty in acquiring a wheelchair due to financial limitations and the failure of the Unified Health System to provide assistive technology resources. These findings corroborate the work by Oliveira et al.,^51^ who identified that mothers of children with CZS are the main caregivers and are responsible for domestic and social demands, facing financial barriers and high social vulnerability. Together, these data reflect the lack of social support and effective public policies to support essential access to assistive technology for children with disabilities in Brazil.

Further, the suspension of health services and development support during the COVID‐19 pandemic led to remote care by health professionals and a rehabilitation routine for mothers and children, strengthening telemedicine. Mothers in this study expressed apprehension and insecurity about performing rehabilitation exercises at home without the direct supervision of physical therapists, similar to Gama et al.^52^ Some mothers reported that even with the guidance of physical therapists, the children did not show noticeable improvements. Vale et al.^53^ also identified regression in the development of children with CZS with exercises performed at home by mothers who learned them in therapy or were guided remotely. Such findings reflect the need for better communication between physical therapists and families about motor prognosis, rehabilitation goals and expectations of telerehabilitation services, which should be established in partnership with the principles of family‐centred and participation‐focused care.^48^

Before COVID‐19, telerehabilitation was used more frequently in high‐income countries.^54^ In low‐ and middle‐income countries (e.g., Brazil), where CERs exclusively developed the majority of rehabilitation interventions, this did not occur. Thus, home‐based programs during the COVID‐19 pandemic that expanded into low‐resource areas may have helped improve the delivery of low‐cost rehabilitation services in natural settings.^48^ However, trained professionals have been required to implement telerehabilitation with few resources, and both professionals and parents have had to demonstrate their commitment to new models of care delivery.^55^

Regarding access to education, mothers whose children attended school perceived a loss in the teaching‐learning process during the pandemic. In a recent study, education professionals felt a lack of training to receive children with CSZ at school; recognized the importance of obtaining health information to ensure children's safety. This was consistent with some perceptions and experiences of mothers in the present study regarding the attitudes of education professionals. Investments in continuing education, structural changes, inclusive equipment and new pedagogical strategies have been suggested to face this scenario and contribute to more inclusive educational spaces for children with disabilities.^56^ Masonbrink and Hurley^57^ further emphasized the need for careful planning for safe and equitable school reopening and strategies that ensure additional support for children with disabilities.

Regarding social isolation, mothers' perceptions of the benefits and drawbacks of social isolation, mirrored existing literature has reported that children's long‐term isolation can impair physical and mental health^58^ or can interfere with the development of consciousness.^59^ Specifically, Vale et al.^53^ showed that a sudden change in the daily lives of children with CZS made it difficult to reestablish or create affective social bonds after the COVID‐19 pandemic. This change was avoided by the mothers in this study. During social distancing, children were surrounded by family protection and familiar places. Therefore, social readaptation after social distancing will require time, empathy and dedication from family members and professionals, aiming to promote the participation of children with CZS.

### 4.1 Study strengths and limitations

This research allowed mothers of children with CZS to express the impact of the COVID‐19 pandemic on the participation of their child. Using photos and focus groups, we extracted details of their life stories by incorporating the mothers as co‐researchers. A strength of this study is that this experience may be one method for empowering mothers of children with CZS to share their stories and improve their confidence in advocating for children's rights. Caregiver empowerment and rights‐oriented advocacy may contribute to the inclusion of children with developmental disorders.^60^ Geographic representation was our main limitation, as mothers were from a single region of Brazil affected by the Zika virus epidemic. Internet instability during remote meetings also presented a challenge, since most mothers lived in the countryside with few resources. Other potential barriers for mothers included domestic demands and the stress of the pandemic, which impacted our ability to assemble, motivate and make mothers feel welcomed to report personal experiences in online meetings. In addition, the sample is relatively small, which may not represent the reality of other children with CZS during the pandemic and will have an impact on the reflections on the transferability of discoveries of these findings. Finally, the presence of the researchers may have introduced acquiescence or reporting bias, though attempts to mitigate these biases included the use of a common set of guiding questions, openness and unconditional positive regard for all experiences that allowed a deeper exploration of the participants' individual contexts, and the use of multiple researchers and member‐checking throughout the research to ensure accuracy and avoid misinterpretation of the mothers' experiences and resulting themes.

### 4.2 Final considerations

This PAR study identified the perceptions of mothers of children with CZS on the impact of the COVID‐19 pandemic on daily life. The photos captured several aspects of the routine of children with CZS. They also highlighted the importance of considering the negative effects of social isolation and preferences of children with CZS, offering school and social assistance to promote participation. The findings of this research can guide changes in the availability of services and support for families of children with CZS in Brazil, reaffirming the purpose of participatory research as a driver of social change.

## AUTHOR CONTRIBUTIONS

Dinara Laiana de Lima Nascimento Dinara Coutinho was involved in the project development, recruitment, data collection, analysis, interpretation of results, manuscript preparation and review. Heather Feldner participated in reviewing and editing the manuscript. Monique L. G. Coelho was involved in the analysis and interpretation of the results. Karoline S. Monteiro was involved in the analysis, interpretation of results, review and editing of the manuscript. Egmar Longo was involved in project guidance, study design, analysis, interpretation of results and review of the manuscript. All authors reviewed the results and approved the final version of the manuscript.

## CONFLICT OF INTEREST STATEMENT

The authors declare no conflict of interest.

## Supporting information

Supplementary information.

## Data Availability

Data are available on request due to privacy/ethical restrictions.

## References

[1] Schiariti V , Longo E , de Campos AC . Impact of congenital Zika virus and COVID‐19 on childhood disability in Latin America. Dev Med Child Neurol. 2021;63(11):1241. 10.1111/dmcn.14998 34651309 PMC8652798

[2] Pergolizzi J , LeQuang JA , Umeda‐Raffa S , et al. The Zika virus: lurking behind the COVID‐19 pandemic? J Clin Pharm Ther. 2021;46:267‐276. 33217046 10.1111/jcpt.13310 PMC7753281

[3] Garcia JV . “No dia em que eu caí ninguém entendeu, porque eu era guerreira”: maternagem e Síndrome Congênita do Vírus Zika em tempos de Covid‐19. Teoria e Cultura. 2022;17(1):105‐117.

[4] Costa F , Sarno M , Khouri R , et al. Emergence of congenital Zika syndrome: viewpoint from the front lines. Ann Intern Med. 2016;164(10):689‐691. 10.7326/M16-0332 26914810 PMC5444536

[5] Molnar Z , Kennedy S . Neurodevelopmental disorders: Risks of Zika vírus during the first trimester of pregnancy. Nat Rev Neurol. 2016;12:315‐316. 10.1038/nrneurol.2016.71 27150532

[6] Ministério da Saúde . Situação epidemiológica da síndrome congênita associada à infecção pelo vírus Zika em 2020. Ministério da Saúde; 2020.

[7] Teixeira GA , Dantas DNA , Carvalho GAFL , Silva A , Lira A , Enders BC . Analysis of the concept of the Zika Virus congenital syndrome. Cien Saude Colet. 2020;25(2):567‐574. 10.1590/1413-81232020252.30002017 32022196

[8] Peiter PA , Pereita R , França I . Análise de dimensões do acesso à saúde das crianças com Síndrome Congênita de Zika (SCZ) na Região Metropolitana do Rio de Janeiro. Saúde Soc. 2020;29(2):1‐14. 10.1590/S0104-12902020200064

[9] Coelho MLG , Campos TNC , Magalhães AG , et al. My child is growing and now? Exploring the environmental needs of children with Congenital Zika Syndrome according to their caregivers' perceptions. Health Expect. 2022;25(6):2828‐2836. 10.1111/hex.13587 36281641 PMC9700161

[10] Carlotti APC , Carvalho WB , Johnston C , et al. Update on the diagnosis and management of COVID‐19 in pediatric patients. Clinics (Sao Paulo). 2020;75:e2353. 10.6061/clinics/2020/e2353 33263635 PMC7688073

[11] Longo E , de Campos AC , Schiariti V . COVID‐19 pandemic: is this a good time for implementation of home programs for children's rehabilitation in low‐ and middle‐income countries? Phys Occup Ther Pediatr. 2020;40:361‐364. 10.1080/01942638.2020.1759947 32408834

[12] Araujo RL , Oliveira GP . Potenciais danos silenciosos da pandemia COVID‐19 em crianças com transtorno do neurodesenvolvimento e paralisia cerebral. Residência Pediátrica; 2020. 10.25060/residpediatr-2020.v10n3-41

[13] Maior CDS . Superação de uma mãe deficiente física no cuidar da filha com paralisia cerebral discinética distônica durante isolamento social no enfrentamento ao coronavírus COVID‐19. Revista Apae Ciência. 2021;16(2):219‐231. 10.29327/216984.16.1-19

[14] Matos SS , Da Silva. ACR . Quando duas epidemias se encontram: a vida das mulheres que têm filhos com a Síndrome Congênita do Zika Vírus na pandemia da COVID‐19. Cadernos De Campo (São Paulo—1991). 2020;29:329‐340. 10.11606/issn.2316-9133.v29isuplp329-340

[15] Cardoso VD , Nicoletti LP , Haiachi MC . Impactos da pandemia do COVID‐19 e as possibilidades de atividades físicas e esportivas para pessoas com deficiência. Rev Bras Ativ Fís Saúde [Internet]. 2023;25:1‐5. 10.12820/rbafs.25e0119

[16] Schiarit V . Os direitos humanos das criancas com deficiência durante emergências de saude: o desafio do COVID‐19. Dev Med Child Neurol. 2020;62(6):E2. 10.1111/dmcn.14528 32277483

[17] Shikako‐Thomas K , Kolehmainen N , Ketelaar M , Bult M , Law M . Promoting leisure participation as part of health and well‐being in children and youth with cerebral palsy. J Child Neurol. 2014;29(8):1125‐1133. 10.1177/0883073814533422 24907136

[18] Peixoto D , Leal B , Ribeiro D , et al. Impact of confinement on the health of children and adolescents during the COVID‐19 pandemic. Act Med Port. 2021;34(4):312‐326. 10.20344/amp.15885 33661728

[19] UN . Convention on the Rights of the Child. United Nations General Assembly; 1989.

[20] UN . Convention on the Rights of Persons with Disabilities. United Nations General Assembly; 2006.

[21] Imms C , Green G , eds. Participation: Optimising Outcomes in Childhood‐Onset Neurodisability. Mac Keith Press; 2020:288.

[22] Feldner HA , Logan SW , Galloway JC . Mobility in pictures: a participatory photovoice narrative study exploring powered mobility provision for children and families. Disabil Rehabil Assist Technol. 2019;14(3):301‐311. 10.1080/17483107.2018.1447606 29522358

[23] Freire P . Pedagogia do Oprimido. Paz e Terra; 2005.

[24] Velez‐Grau C . Using photovoice to examine adolescents' experiences receiving mental health services in the United States. Health Promot Int. 2019;34(5):912‐920. 10.1093/heapro/day043 29986026

[25] Wang CC , Redwood‐Jones YA . Photovoice ethics: perspectives from flint photovoice. Health Educ Behav. 2001;28:560‐572. 10.1177/109019810102800504 11575686

[26] Call‐Cummings M , Hauber‐Ozer M . Virtual photovoice: methodological lessons and cautions. Qual Rep. 2021;26(10):3214‐3233. 10.46743/2160-3715/2021.4971

[27] Dassah E , Aldersey HM , Norman KE . Photovoice and persons with physical disabilities: a scoping review of the literature. Qual Health Res. 2017;27(9):1412‐1422. 10.1177/1049732316687731 28682708

[28] Greco V , Lambert HC , Park M . Being visible: PhotoVoice as assessment for children in a school‐based psychiatric setting. Scand J Occup Ther. 2016;24(3):222‐232. 10.1080/11038128.2016.1234642 27665933

[29] Jack SM , Phoenix M . Qualitative health research in the fields of developmental medicine and child neurology. Dev Med Child Neurol. 2022;64(7):830‐839. 10.1111/dmcn.15182 35156198

[30] King G, McPherson AC, Kingsnorth S, Gorter JW. The transformative nature of residential immersive life skills programs: integrating findings from a five‐year prospective study of program opportunities, youth experiences, and outcomes. Int J Environ Res Public Health. 2022;19(23):15865. 10.3390/ijerph192315865 36497940 PMC9740383

[31] Wang C , Burris MA . Photovoice: concept, methodology and use for participatory needs assessment. Health Educ Behav. 1997;24(3):369‐387. 10.1177/109019819702400309 9158980

[32] Krutt H , Dyer L , Arora A , Rollman J , Jozkowski AC . PhotoVoice is a feasible method of program evaluation at a center serving adults with autism. Eval Program Plann. 2018;68:74‐80. 10.1016/j.evalprogplan.2018.02.00310.1016/j.evalprogplan.2018.02.003 29494812

[33] Finger D , Souza JB , Berlezi GD , Zanettini A . Music, health, nursing: family perception on the coral corner in child development. J Nurs. 2017;11(8):3251‐3327.

[34] Neves AT , Donaduzzi DSS , Santos RP , et al. O processo de inclusão de crianças com necessidades especiais de saúde na educação infantil. Revista de Enfermagem da UFSM. 2017;7(3):374‐387.

[35] Walker A , Colquitt G , Elliott S , Emter M, Li L. Using participatory action research to examine barriers and facilitators to physical activity among rural adolescents with cerebral palsy. Disabil Rehabil. 2020;42(26):3838‐3849. 10.1080/09638288.2019.1611952 31088164

[36] Liebenberg L . Pensando criticamente sobre Photovoice: Alcançando Empoderamento e Mudança Social. Jornal Internacional de Métodos Qualitativos. 2018;17:1‐9. 10.1177/1609406918757631journals.sagepub.com/home/ijq

[37] Eisen I , Cunningham BJ , Campbell W . Conducting participatory photography with children with disabilities: a literature review. Disabil Rehabil. 2017;41(16):1943‐1954. doi:10,1080/09638288.2018.1457089 10.1080/09638288.2018.1457089 29587538

[38] Leal LA , Henriques SH . Guia norteador para condução de grupo focal na identificação de competências gerenciais: Relato de experiência. New Trends Qual Res. 2021;8:890‐897. 10.36367/ntqr.8.2021.890-897

[39] Sposito AMP , Garcia‐Schinzari NR, Mitre RMA, Pfeifer LI, Lima RAG, Nascimento LC. The best of hospitalization: contributions of playing to cope with chemotherapy. Av Enferm. 2018;36(3):328‐337. 10.15446/av.enferm.v36n3.61319

[40] Reichenberger V , Smythe T , Hameed S , et al. Participatory visual methods with caregivers of children with congenital Zika syndrome in Colombia: a case study. Wellcome Open Res. 2022;7:107. 10.12688/wellcomeopenres.17529.1 37928610 PMC10620479

[41] Martins FR , Franco SEJ , Santos ACC , Guimarães AL , Oliveira PR . Repercussões emocionais em mães de crianças com microcefalia em decorrência do Zika Vírus. Res Soc Dev. 2021;10(6):e18410615444. 10.33448/rsd-v10i6.15444

[42] Santos D , Prado LOM , Silva R , Silva E , Cardoso L , Oliveira C . Sensitizing mothers of children with microcephaly in promoting the health of their children. Rev Esc Enferm USP. 2019;53:03491. 10.1590/S1980-220X2018022903491 31433020

[43] Bates MJ , Ardrey J , Mphwatiwa T , Squire SB , Niessen LW . Enhanced patient research participation: a photovoice study in Blantyre Malawi. BMJ Support Palliat Care. 2018;8:171‐174. 10.1136/bmjspcare-2017-001439 PMC5969331 29420196

[44] Imms C , Reilly S , Carlin J , Dodd K . Diversity of participation in children with cerebral palsy. Dev Med Child Neurol. 2008;50:363‐369. 10.1016/j.ejpn.2008.03.005 18355337

[45] Shikako‐Thomas K , Majnemer A , Law M , Lach L . Determinants of participation in leisure activities in children and youth with cerebral palsy: systematic review. Phys Occup Ther Pediatr. 2008;28(2):155‐169. 10.1080/01942630802031834 18846895

[46] Maria‐Mengel MRS , Linhares MBM . Risk factors for infant developmental problems. Rev Lat Am Enfermagem. 2007;15(15):837‐842. 17934592 10.1590/s0104-11692007000700019

[47] Santos LS , Barbosa ASS , Santana AFSG , et al. A participação da família no trabalho de reabilitação da criança com microcefalia. Caderno de Graduação—Ciências Biológicas E Da Saúde—UNIT—ALAGOAS. 2018;4(2):189.

[48] Longo E , de Campos AC , Palisano RJ . Let's make pediatric physical therapy a true evidence‐based field! Can we count on you? Braz J Phys Ther. 2019;23(3):187‐188. 30420270 10.1016/j.bjpt.2018.10.011 PMC6531638

[49] Sá FE . Parental needs in the care for children with Zika virus‐induced microcephaly. Revista Brasileira de Promoção da Saúde. 2017;30(4):1‐10. 10.5020/18061230.2017.6629

[50] Albuquerque MSV , Lyra TM , Melo APL , et al. Access to healthcare for children with Congenital Zika Syndrome in Brazil: perspectives of mothers and health professionals. Health Policy Plan. 2019;34:499‐507. 10.1093/heapol/czz059 31369667 PMC6788207

[51] Oliveira MC , Moreira RCR , Lima MM , Melo RO . Vivências de mães que tiveram filhos com microcefalia. Revista Baiana de enfermermagem. 2018;32:1‐11. 10.18471/rbe.v32.26350

[52] Gama GL , Silva BM , Silva BM , et al. Mental health and burden in mothers of children with congenital Zika syndrome during COVID‐19 pandemic. Rev Bras Saúde Matern Infant. 2021;21(suppl 2):S481‐S490. 10.1590/1806-9304202100S200009

[53] Vale PRLF , Alves DV , Amorim RC , et al. Rosette of care for children with zika congenital syndrome: caring attitudes of relatives. Esc Anna Nery. 2020;24(3):1‐8. 10.1590/2177-9465-EAN-2019-0268

[54] Levac D , Glegg SMN , Camden C , Rivard LM , Missiuna C . Best practice recommendations for the development, implementation, and evaluation of online knowledge translation resources in rehabilitation. Phys Ther. 2015;95(4):648‐662. 25301966 10.2522/ptj.20130500

[55] Alsem MW , Verhoef M , Braakman J , et al. Parental empowerment in paediatric rehabilitation: exploring the role of a digital tool to help parents prepare for consultation with a physician. Child Care Health Dev. 2019;45:623‐636. 10.1111/cch.12700 31276605 PMC7171983

[56] Avelino MOA , Ferraz PCS. Educação Inclusiva: Olhar dos Profissionais Sobre as Crianças Com Síndrome Congênita do Zika Vírus na Gerência Regional de Educação Cajazeiras e Pirajá: um Estudo Transversal. Rev Bras Ed Esp. 2021;27:251‐268. 10.1590/1980-54702021v27e0056

[57] Masonbrink AR , Hurley E . Advocating for children during the COVID‐19 school closures. Pediatrics. 2020;146(3):e20201440. 10.1542/peds.2020-1440 32554517

[58] Patel K . Mental health implications of COVID‐19 on children with disabilities. Asian J Psychiatry. 2020;54:102273. 10.1016/j.ajp.2020.102273 PMC7330593 32653852

[59] Kotzky K , Allen JE , Robinson LR , et al. Depressive symptoms and care demands among primary caregivers of young children with evidence of congenital Zika virus infection in Brazil. J Dev Behav Pediatr. 2019;40(5):344‐353. 30921104 10.1097/DBP.0000000000000666 PMC6579666

[60] Slamka ZS , Tekola BT , Hoekstra R , Hanlon C. The role of advocacy and empowerment in shaping service development for families raising children with developmental disabilities. Health Expect. 2022;25(4):1882‐1891. 10.1111/hex.13539 35644908 PMC9327816

