# Supplementary material for: The burden of global outbreaks: Photos of the daily lives of children with congenital Zika syndrome during the COVID‐19 pandemic
Source: Health Expect. 2023 Aug 18;26(6):2500–13. doi: 10.1111/hex.13717 (PMC10632653; doi:10.1111/hex.13717)
Supplement: Supplementary file 1 — Supplementary information. [file HEX-26--s001.docx]

**Supplementary Material**

1. ***Preferences for participation***

“I think he likes it when we let him do something.” (Photo 1, Júlia, mother of Daniel, 5 years old, GMFCS=IV).

“He dislikes being alone. He does not want to be alone at any time. If he is alone, he cries, he complains... that is why I posted this picture of him in bed; that is the only time we know he does not like it. Every moment of the day, we try to involve him with the family, at lunchtime, at dinner... We always put him close; wherever we are, he is with us (Photo 2, Daniela, Leonardo's mother, 5 years old, GMFCS =V).”

Photo 1: Daniel playing with water, one of his favorite activities


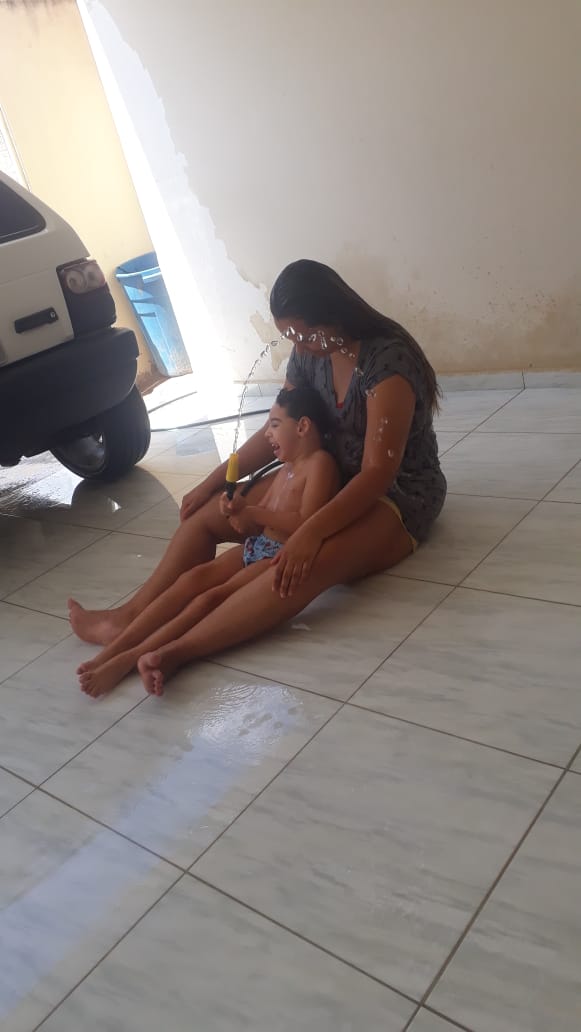


Source: Julia, Daniel's mother (2020)

Photo 2: Leonardo alone, lying on his mother's bed

.
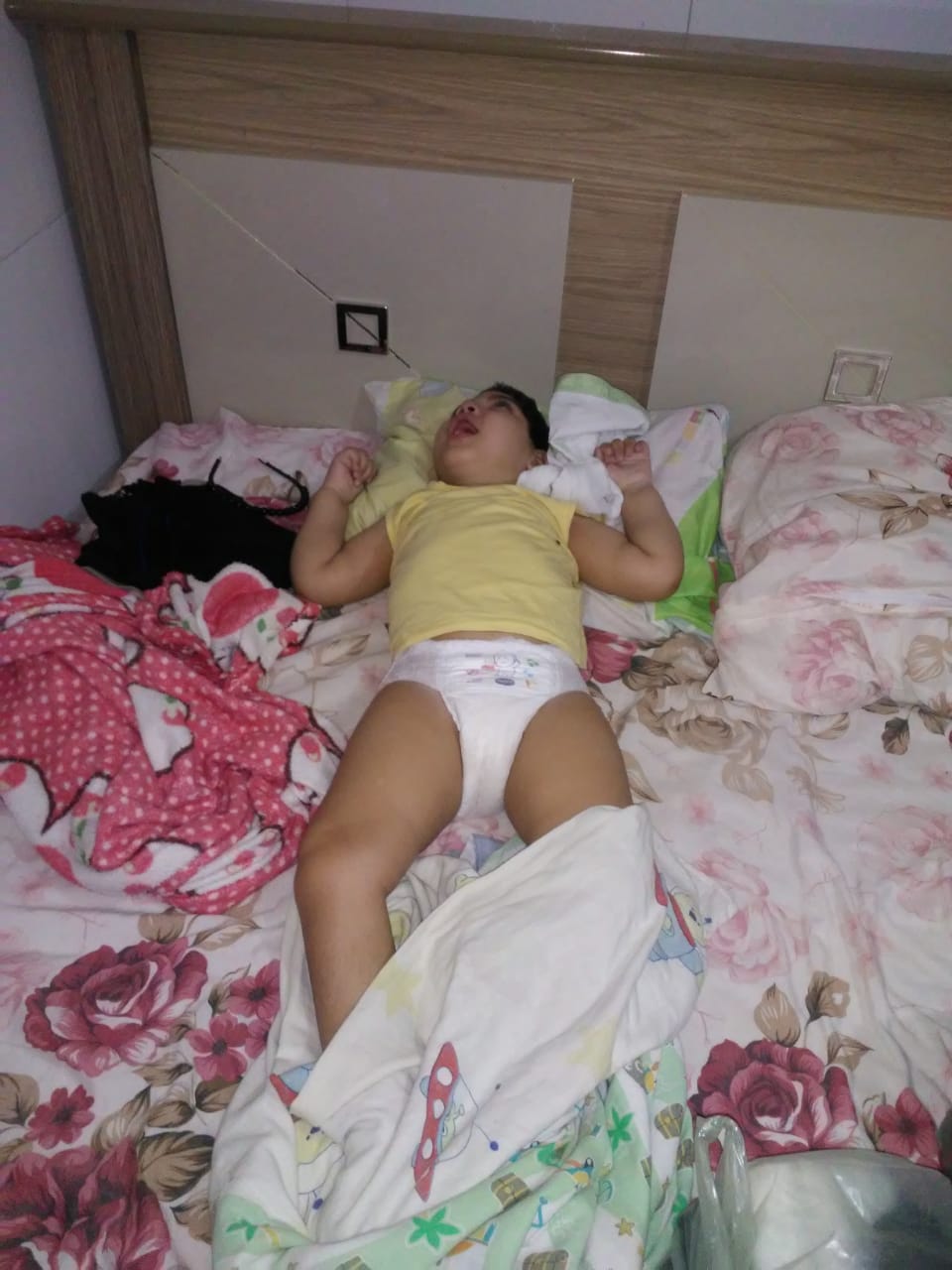


Source: Daniela, Leonardo's mother (2020)

“In this photo, he is alone. He does not like to be alone for too long. In the photo, he is happy because he saw I was coming back to get him. (Photo 3, Júlia, Daniel's mother, 5 years old, GMFCS=IV).”

Photo 3: Daniel happy to see his mother.


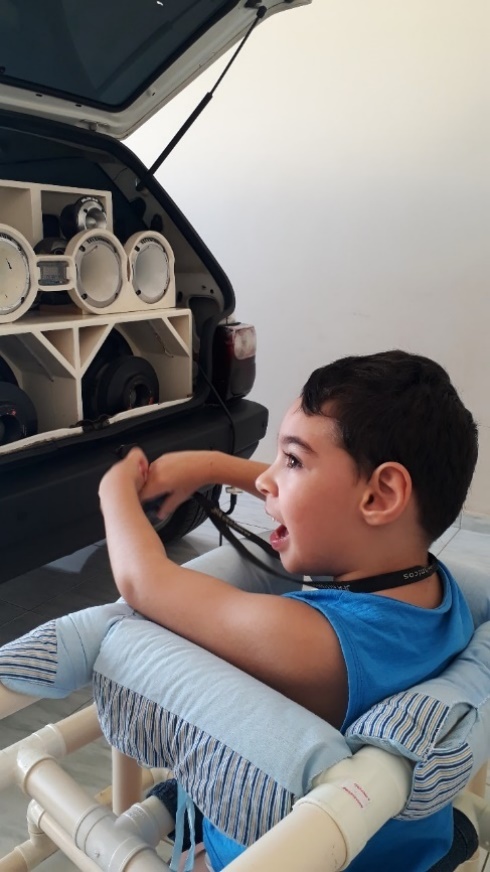


Source: Julia, Daniel's mother (2020)

***2. Family relationships***

“The company of his father, his sister, and I is what makes him have a better development; it is what makes him play and smile” (Photo 4, Daniela, Leonardo's mother, 5 years old, GMFCS =V).”

Photo 4: Leonardo receiving affection from his sister.


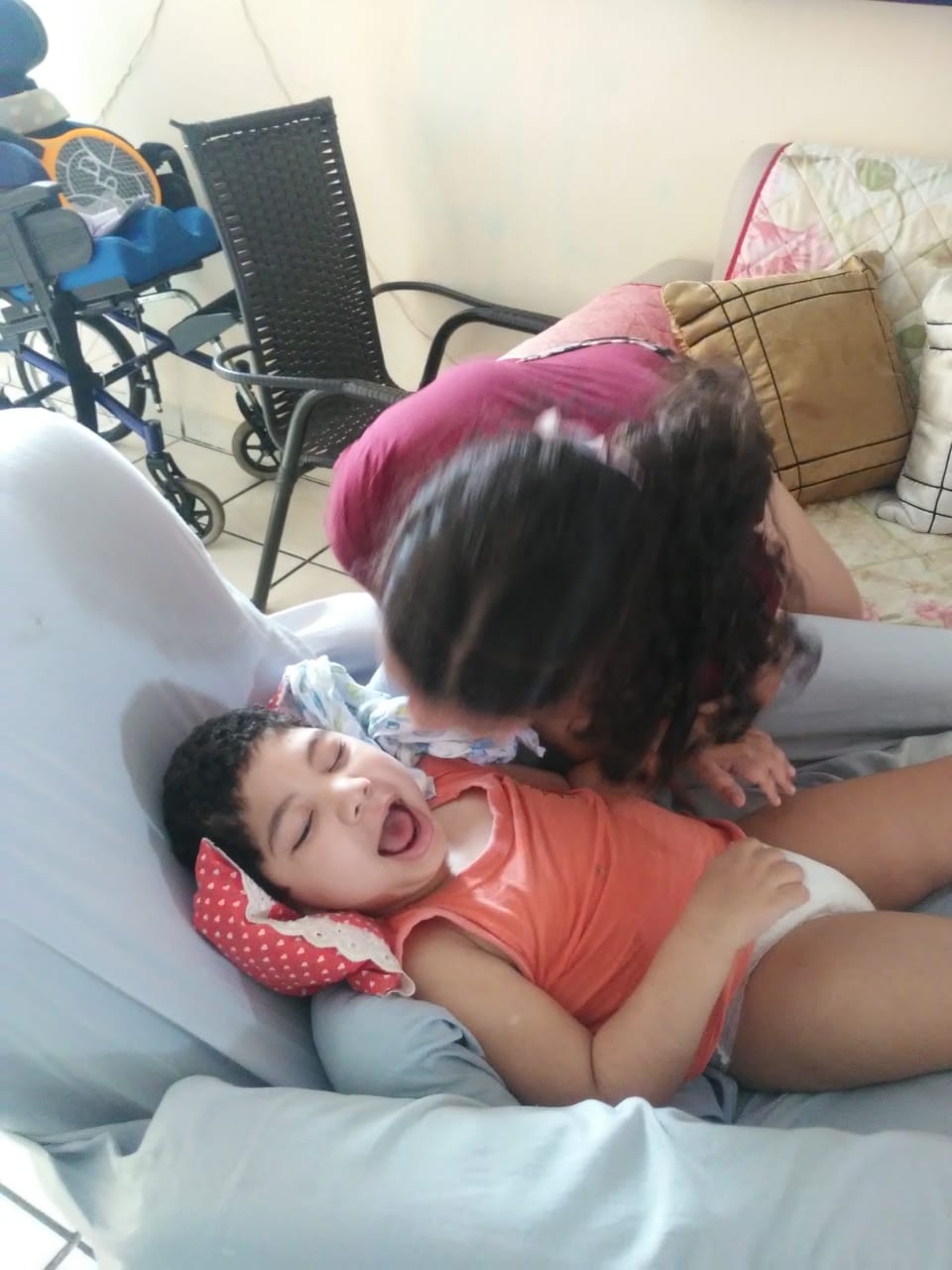


Source: Daniela, Leonardo's mother (2022).

“In this moment her father was leaving to work, and I took the picture. She was crying because her father left the house. I think she imagines he will not come back home.” (Photo 5, Marta, mother of Clara, 5 years old, GMFCS=5)”.

Photograph 5: Clara crying when she saw her father leaving for work.


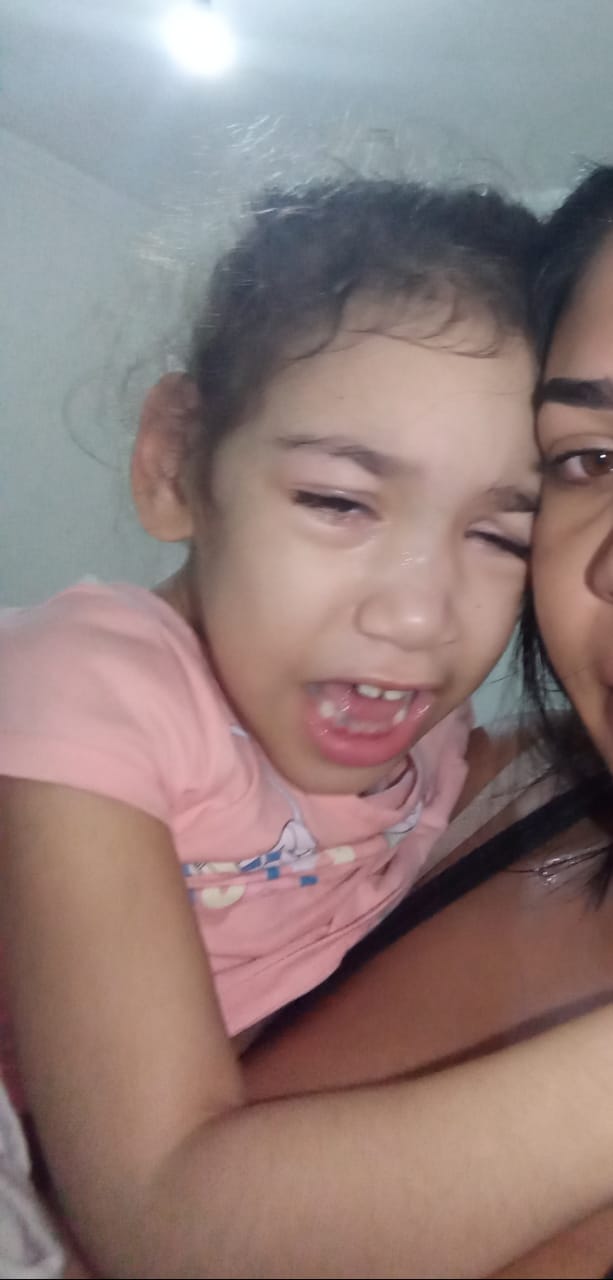


Source: Marta, mother of Clara (2022).

“He feels loved and responds to affection with this smile, with his look (Photo 6, Mother of Mateus, 2 years old, GMFCS=V)”.

.Photo 6: Mateus smiling at his mom.


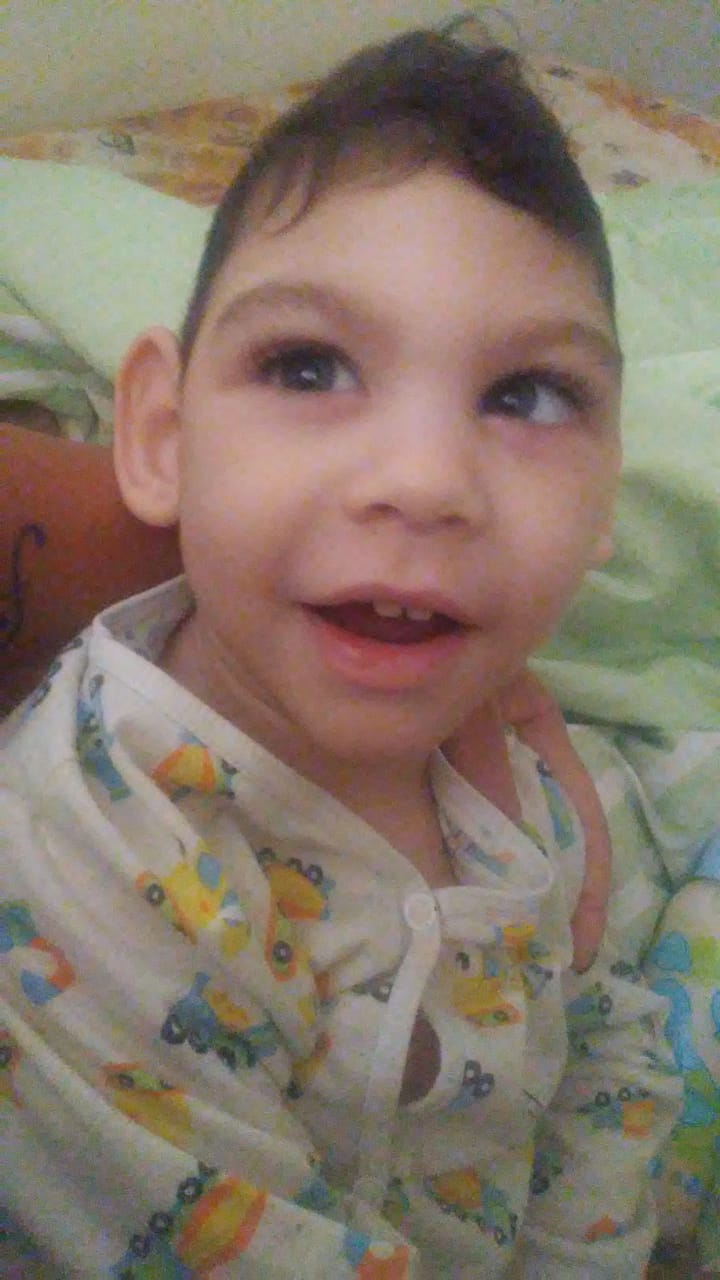


Source: Patricia, mother of Mateus (2020).

“This photo is when it was time to sleep, and she wants to keep talking. She is expressing a feeling of wanting attention, for me to pay attention to her, to play around (Photo 7, Mother of Ingrid, 4 years old, GMFCS=V)”.

Photo 7: Ingrid trying to talk.


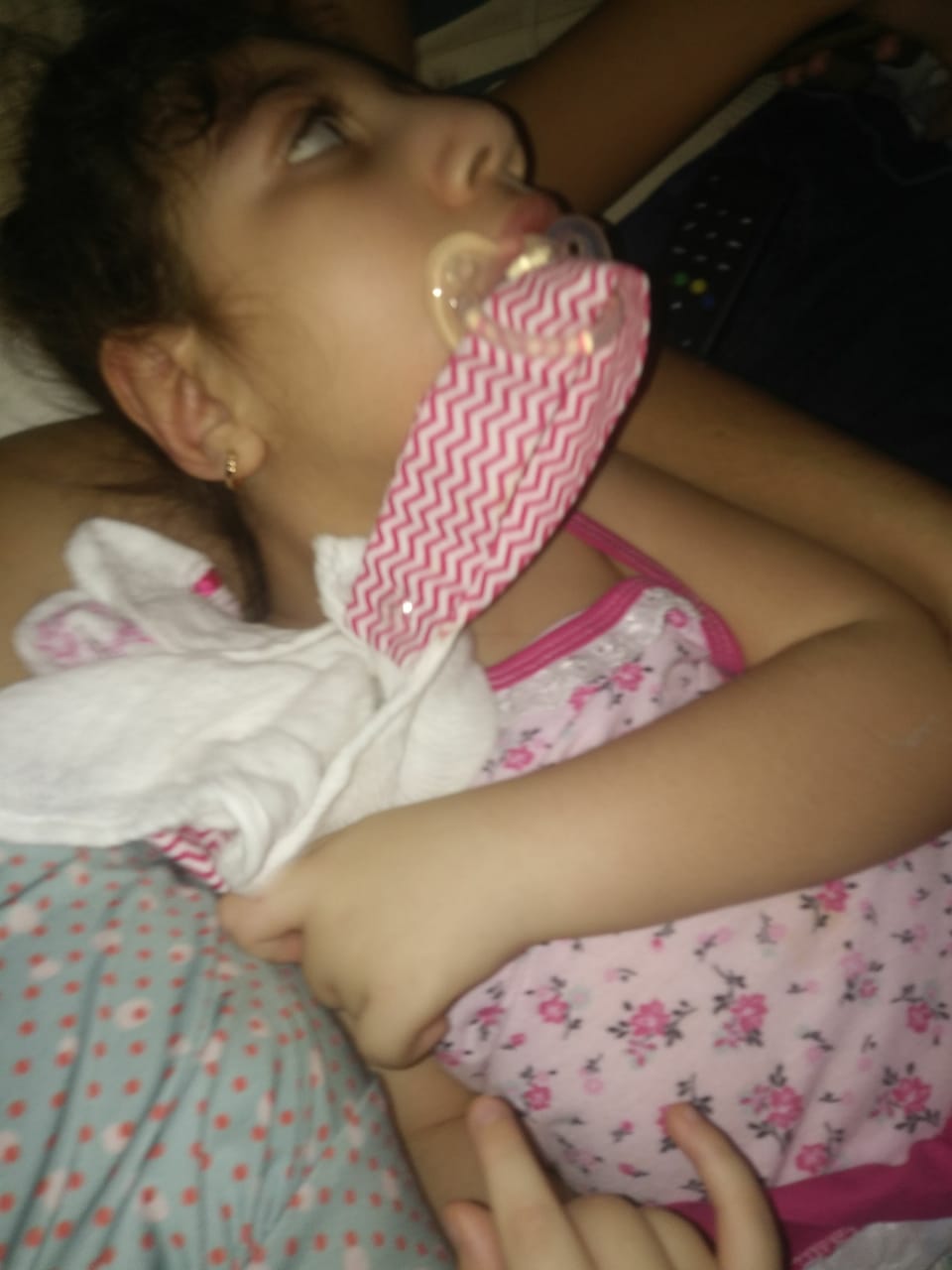


Source: Irece, mother of Ingrid (2022).

“Her little cousin feeding her is what makes her happy, her cousin coming to our house and feeding her” (Photo 8, Irece, mother of Ingrid, 4 years old, GMFCS=V).

Photo 8: Ingrid's cousin giving her food.


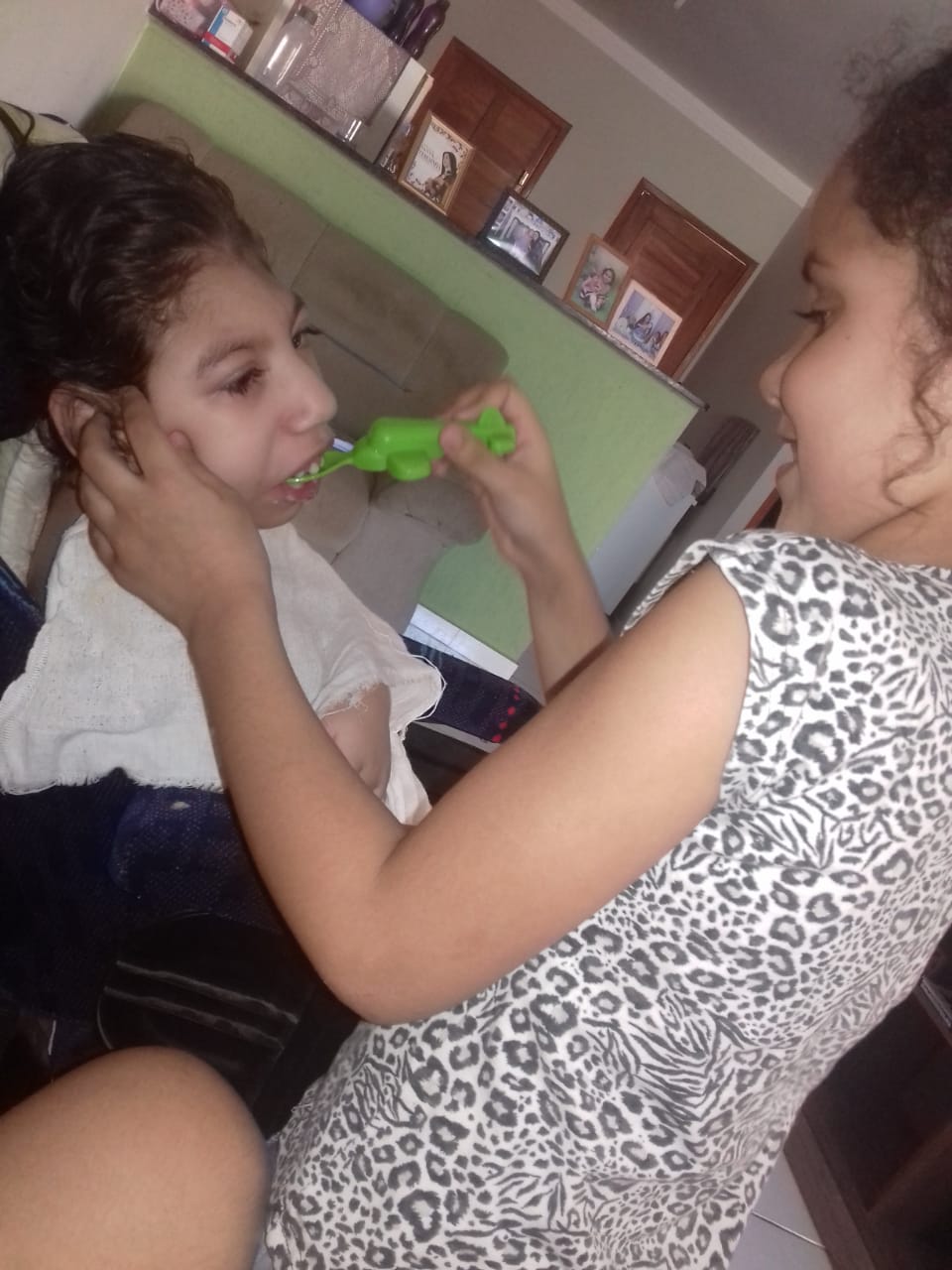


Source: Irece, mother of Ingrid (2022).

“He shows us a happy feeling for being with family, that feeling of peace, of tranquility. The photo demonstrates the reality of his life” (Photo 9: Daniela, mother of Leonardo, 5 years old, GMFCS=V).

Photo 9: Leonardo, watching tv and enjoying the comfort of his home.


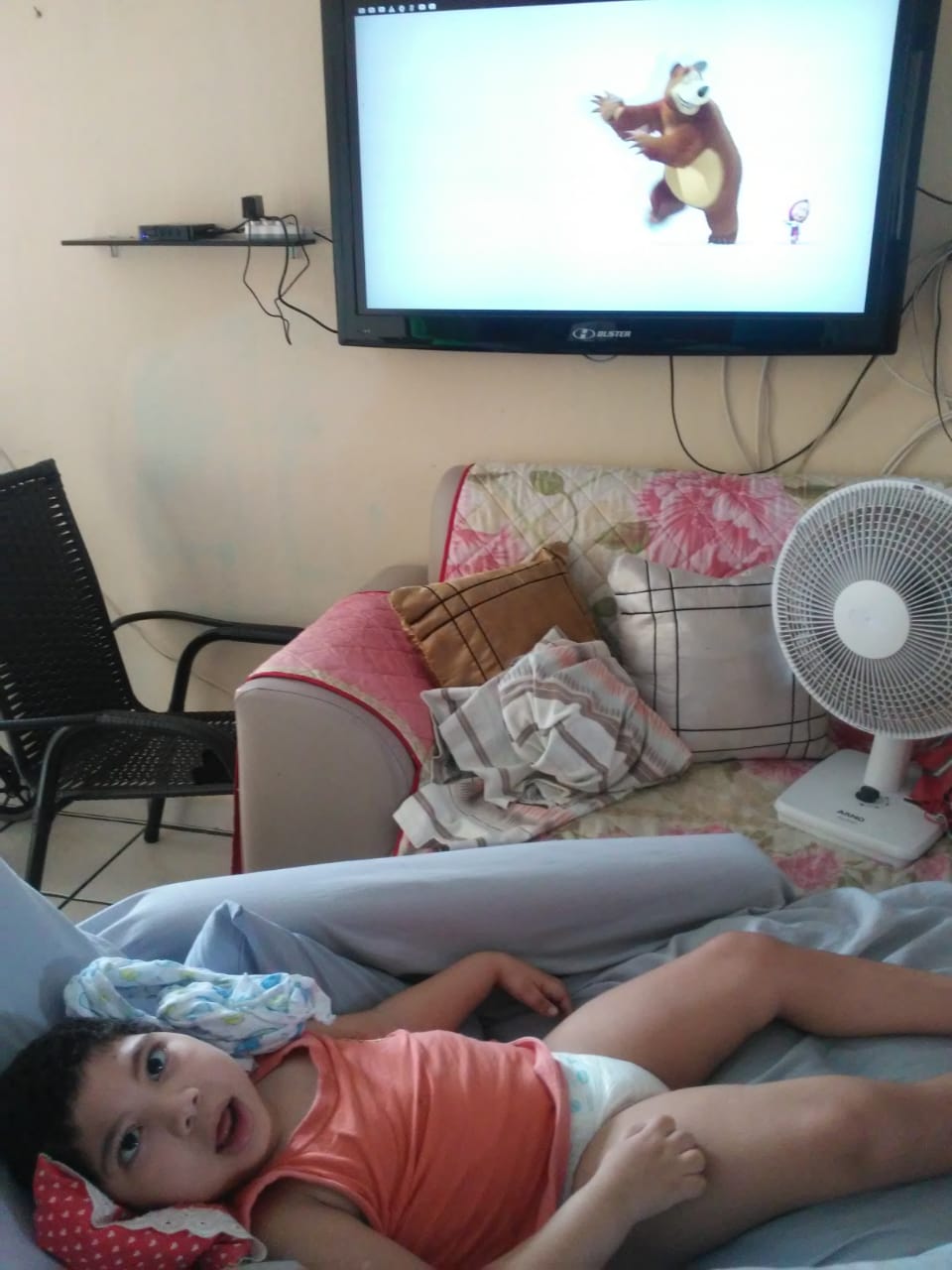


Source: Daniela, Leonardo's mother (2020).

***-Category 3: access to health and development support***

“Now, in the pandemic, the treatment is more difficult because she does not have physical therapy, which helps a lot (Photo 10, Mother of Clara, 5 years old, GMFCS=5).”

Photo 10: Clara during her Physiotherapy.


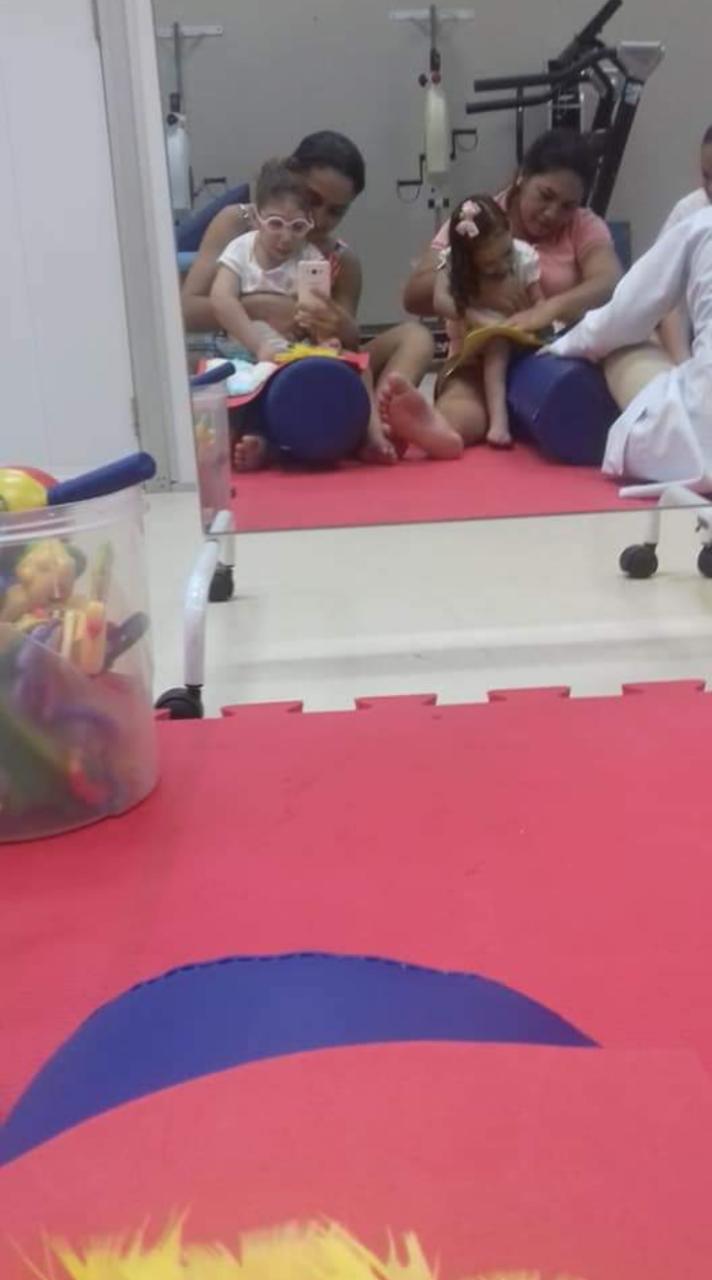


Source: Marta, mother of Clara (2022).

“His leg is very bent. It is one on top of the other. He tries to move his leg but cannot, maybe because of the leg weight and because the upper leg is hurting the bottom leg. It appears [the orthosis] is weighing him down, bothering him, squeezing him... like he wanted to take that thing off (Photo 11, Patrícia, mother of Mateus, 2 years old, GMFCS=V)”.

Photo 11: Mateus with his orthosis


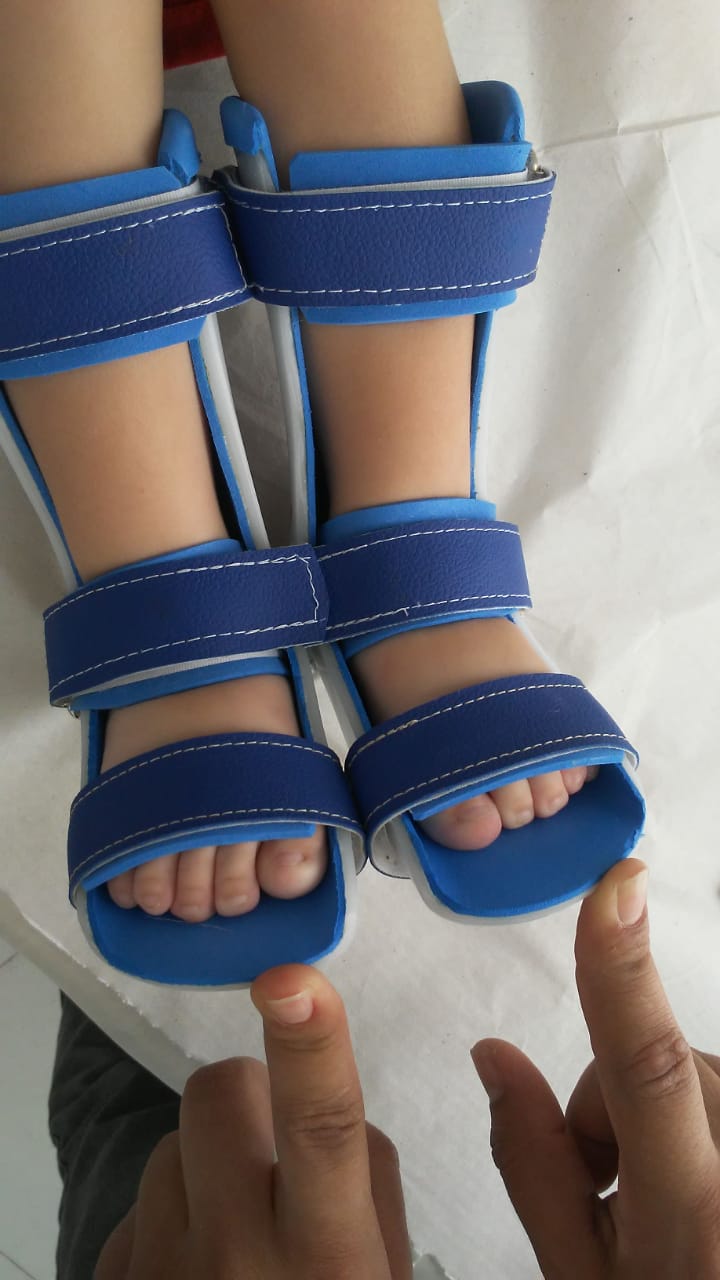


Patricia, mother of Mateus (2020).

“Almost every day she went to physical therapy using the City Hall car (Photo 12, Mother of Ingrid, 4 years old, GMFCS=5, Irece)”

Photo 12: Ingrid going to Physiotherapy in the City Hall car.


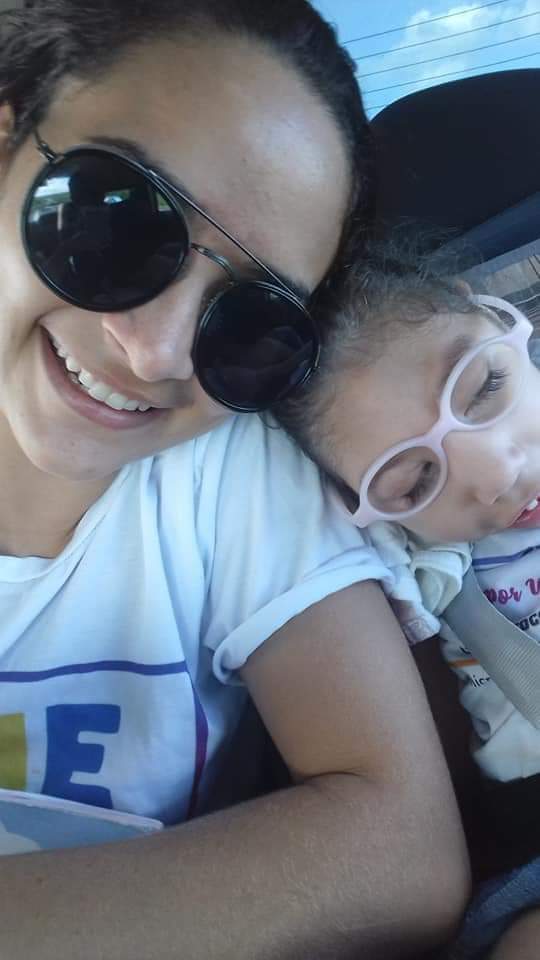


Source: Irece, mother of Ingrid (2022).

“Before the pandemic, we would go out a lot to look for treatments, physical therapy, medical appointments, and every benefit he is entitled to...so every week we had something to do and many exhausting trips...however, with the pandemic, the development regressed a lot. He can no longer control his neck due to a lack of physical therapy. Previously, he could take a few steps; he would turn around more. Now, what he has gained, he has already lost... his leg is atrophying a lot and is very crooked compared to the other.” (Boy, two years old, GMFCS = V, Patrícia (Photo 13, Patrícia, mother of Mateus, 2 years old , GMFCS=V)”.

Photo 13: Mateus using the armrest to keep his neck steady.


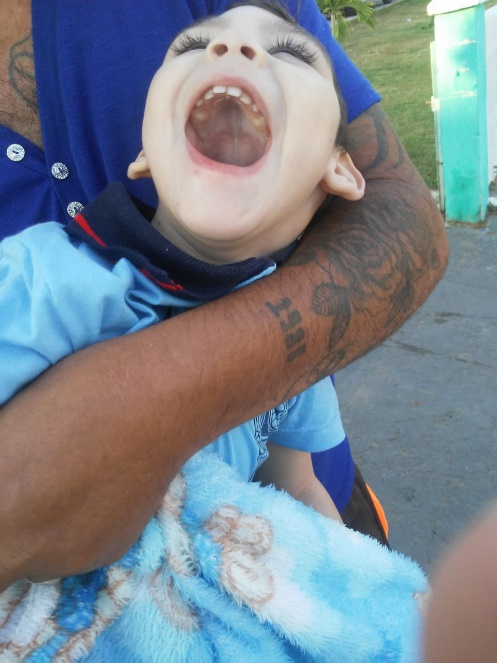


Patricia, mother of Mateus (2020).

***- Category 4: access to education***

“I enrolled her in a daycare center, but she stayed less than a month because they put her in a class of two-year-olds. [The children] cried a lot; she would get irritated and could not adapt. I noticed that the teacher did everything in her power for her to stay at school. She would call and get in touch to convince me to take her to school. On the other hand, the principal did not seem to mind the presence of my daughter and would not even bother to prepare the school to receive her” (Photo 14, Alice's mother, 5 years old, GMFCS= V).

Photo 14: Alice going to school.


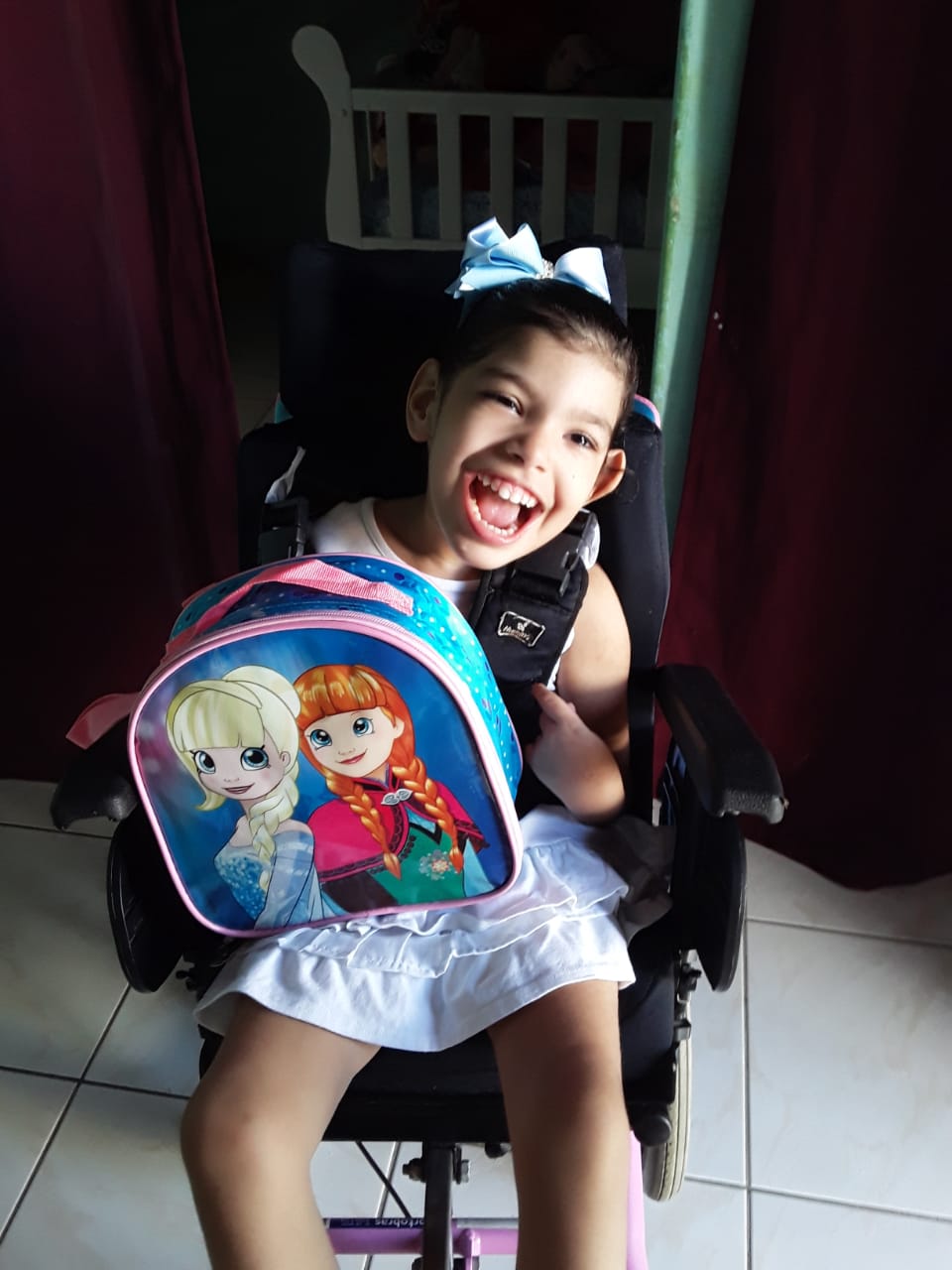


Source: Luísa, Alice's mother.

***-Category 5: social isolation***

“We used to go out a lot to physical therapy, medical appointments, and could not enjoy our time together. You can see that she was happy and smiling to be with me. (Photo 15, Irece, mother of Ingrid, 4 years old, GMFCS=5).”

Photo 15: Irece and Ingrid enjoying their moment together.


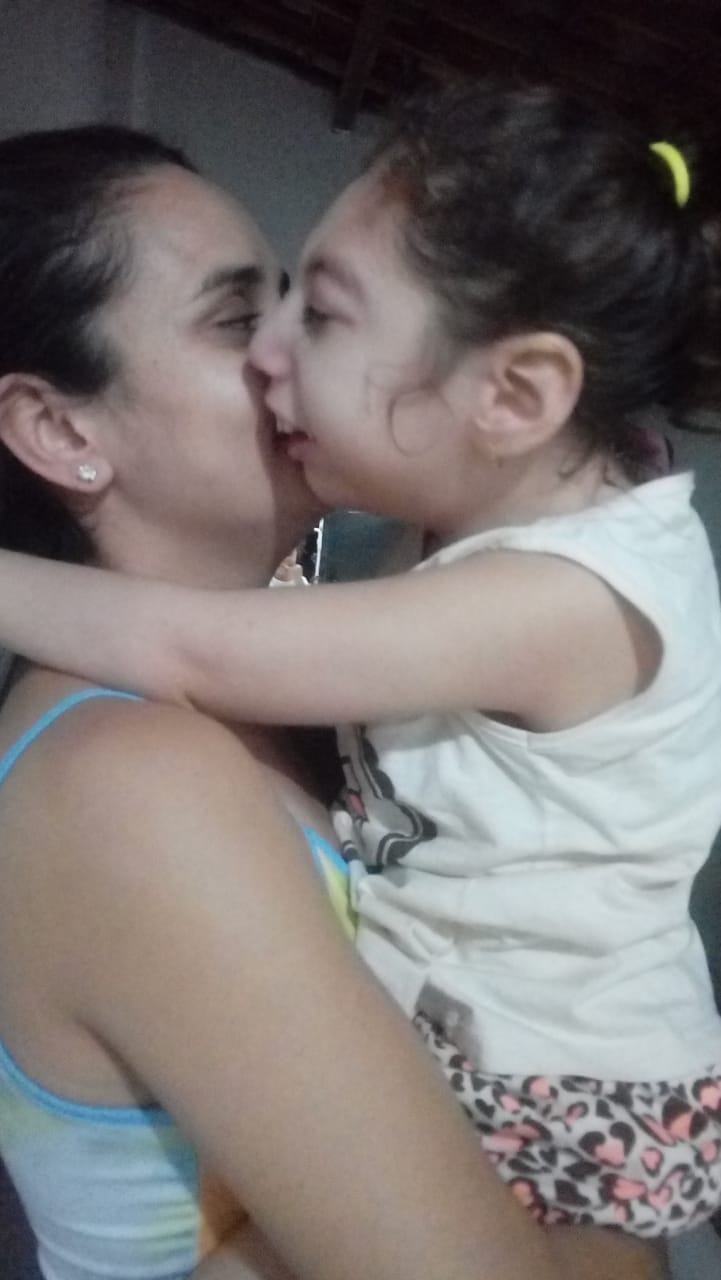


Source: Irece, mother of Ingrid (2022).

“With the pandemic, he has more time to enjoy at home, play, do physical therapy (Photo 16, Júlia, Daniel's mother, 5 years old, GMFCS=IV).”

Photo 16: Daniel bathing in the pool with his mother.


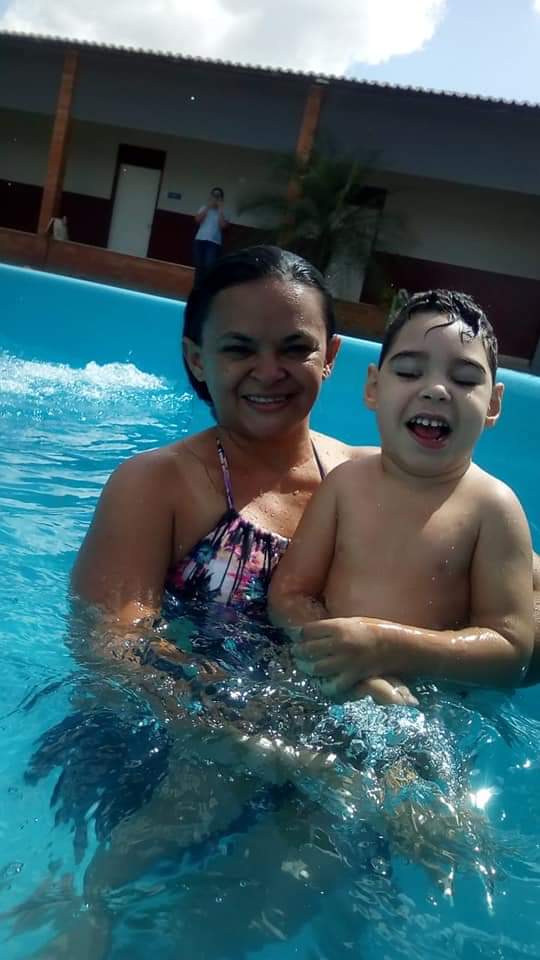


Source: Julia, Daniel's mother (2020)

“The physical therapists gave us some booklets with guidelines, and he was being accompanied through video call on WhatsApp. They would send exercises that we would do at home (Photo 17, Daniel’s mother, 5 years old, GMFCS=IV).”

Photo 17: Daniel doing his physical therapy at home, with the help of his mother.
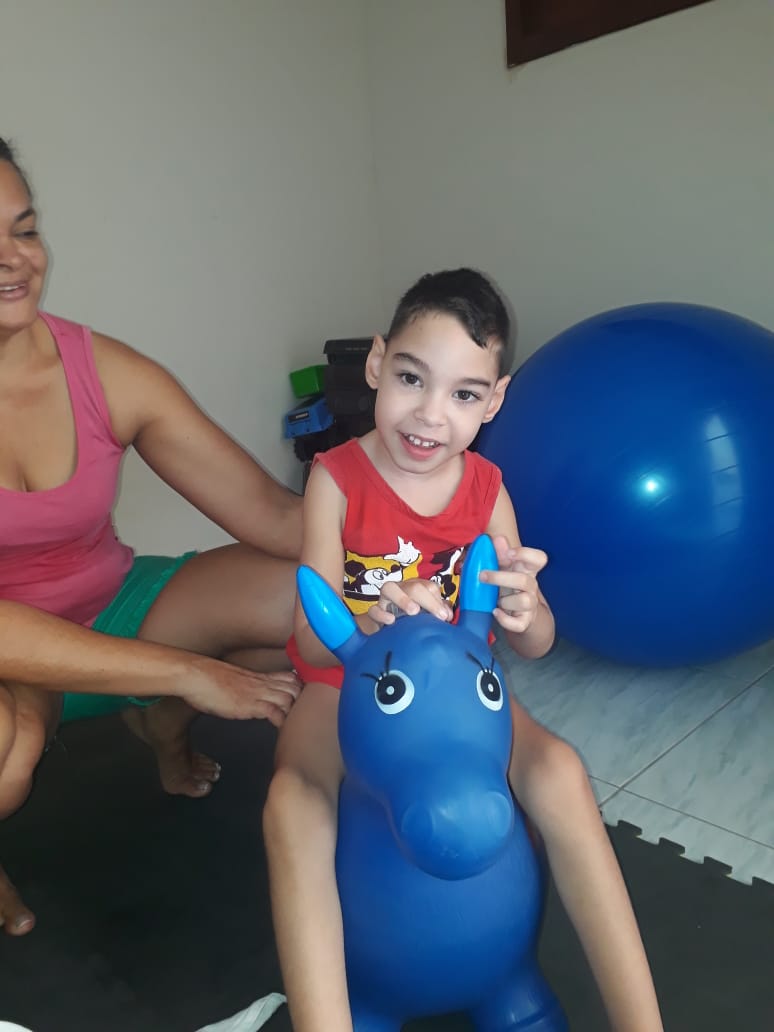


Source: Julia, Daniel's mother (2020)

“When the pandemic is over, I think it will be difficult for her to return to her routine with her tasks. Lately, she dislikes going out or interacting with other people. Nowadays, she likes to stay in her corner... before, she used to like to go for walks (Photo 18, Luísa, Alice's mother, 5 years, GMFCS= V).”

Photo 18: Alice on her mother's lap.


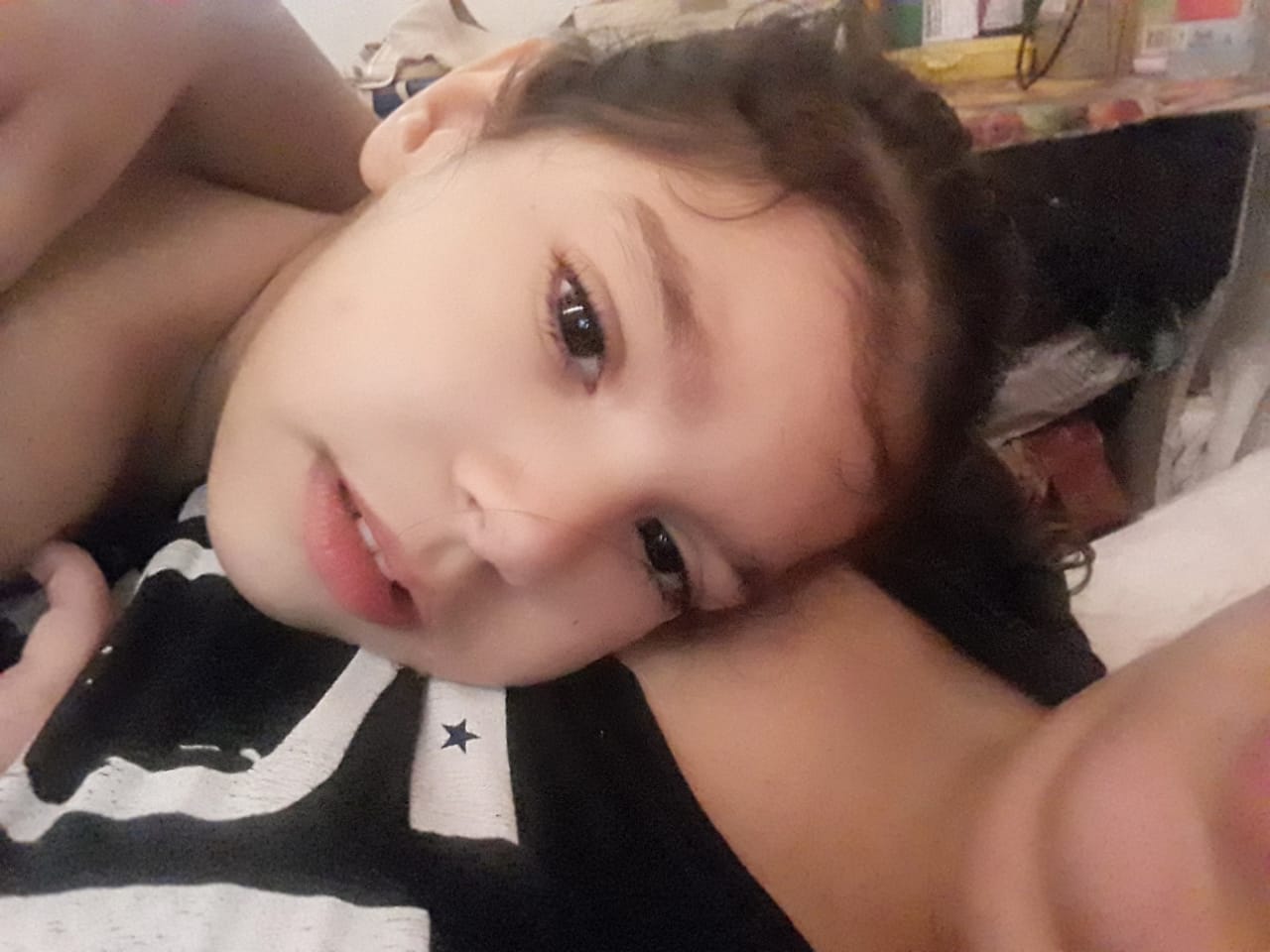


Source: Luísa, Alice's mother.

““His life became more difficult in this daily isolation because he used to go out a lot, and we would take him everywhere we went... Now he only has contact with his family, the three of us at home together with him... I think that was the part he felt the most: he would go out with us a lot, and now we are trapped inside the house. When we leave, it is one or the other, but he remains inside the house. So, for him, I think this isolation part that he needed to stay home more, unable to leave or have fun, made him sadder. I think his sadness is clear when we need to go out (Photo 19, Daniela, Leonardo's mother, 5 years old, GMFCS=V).”

Photo 19: Leonardo sitting, unable to leave the house.


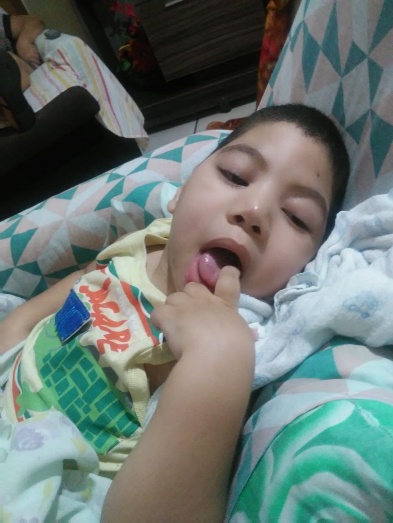


Source: Daniela, Leonardo's mother (2020).

“What made his life easier in this pandemic was having a wheelchair. In the past, he was trapped in a chair, on the couch, or on the bed. Now, even inside the house, it is easy for us to walk him around… Now wherever I go, I can take him; wherever his father is, he can take him close, and his sister also takes him wherever she needs to go. So, I think the wheelchair favored his mobility” (Photo 20, Daniela, Leonardo's mother, 5 years old, GMFCS=V).”

Photo 20: Leonardo sitting in his wheelchair.


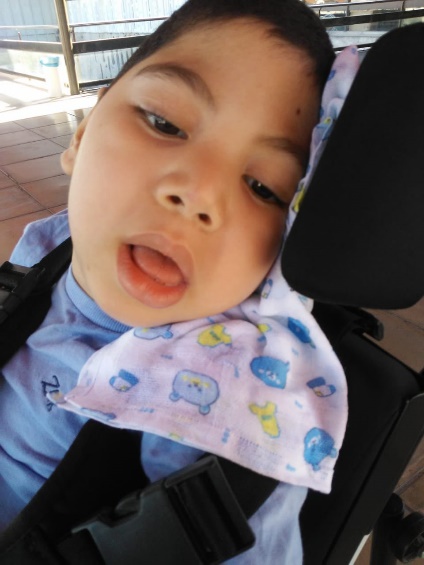


Source: Daniela, Leonardo's mother (2020).
